# Enhanced protein degradation by black soldier fly larvae (*Hermetia illucens* L.) and its gut microbes

**DOI:** 10.3389/fmicb.2022.1095025

**Published:** 2023-01-10

**Authors:** Yongqiang Yu, Jia Zhang, Fengling Zhu, Mingxia Fan, Jinshui Zheng, Minmin Cai, Longyu Zheng, Feng Huang, Ziniu Yu, Jibin Zhang

**Affiliations:** ^1^State Key Laboratory of Agricultural Microbiology, National Engineering Research Center of Microbial Pesticides, College of Life Science and Technology, Huazhong Agricultural University, Wuhan, China; ^2^Hubei Hongshan Laboratory, Wuhan, China; ^3^Renmin Hospital of Wuhan University, Wuhan, China; ^4^Hubei Key Laboratory of Agricultural Bioinformatics, Huazhong Agricultural University, Wuhan, China

**Keywords:** black soldier fly larvae, protein synergistic degradation, 16S rDNA sequencing, ITS1 sequencing, artificial diet

## Abstract

Black soldier fly larvae (BSFL) can convert a variety of organic wastes into biomass, and its gut microbiota are involved in this process. However, the role of gut microbes in the nutrient metabolism of BSFL is unclear. In this study, germ-free BSFL (GF) and gnotobiotic BSFL (GB) were evaluated in a high-protein artificial diet model. We used 16S rDNA sequencing, ITS1 sequencing, and network analysis to study gut microbiota in BSFL that degrade proteins. The protein reduction rate of the GB BSFL group was significantly higher (increased by 73.44%) than that of the GF BSFL group. The activity of gut proteinases, such as trypsin and peptidase, in the GB group was significantly higher than the GF group. The abundances of different gut microbes, including *Pseudomonas* spp., *Orbus* spp. and *Campylobacter* spp., were strongly correlated with amino acid metabolic pathways. *Dysgonomonas* spp. were strongly correlated with protein digestion and absorption. *Issatchenkia* spp. had a strong correlation with pepsin activity. *Campylobacter* spp., *Pediococcus* spp. and *Lactobacillus* spp. were strongly correlated with trypsin activity. *Lactobacillus* spp. and *Bacillu*s spp. were strongly correlated with peptidase activity. Gut microbes such as *Issatchenkia* spp. may promote the gut proteolytic enzyme activity of BSFL and improve the degradation rate of proteins. BSFL protein digestion and absorption involves gut microbiota that have a variety of functions. In BSFL the core gut microbiota help complete protein degradation. These results demonstrate that core gut microbes in BSFL are important in protein degradation.

## 1. Introduction

Insect guts often harbor microbial communities (Engel and Moran, 2013). Insect gut microbiota can have important symbiotic functions such as providing host nutrition and promoting digestion (Schmidt and Engel, 2021). The main role of gut bacteria is amino acid biosynthesis, followed by protein degradation and energy metabolism (Jing et al., 2020).

Larvae of the black soldier fly (BSFL) can degrade a variety of organic substances, including livestock manure, agricultural waste, food waste, cyanobacteria, and pulp waste (Gold et al., 2018; Niero et al., 2022; Peguero et al., 2022; Zhang et al., 2022; Lu, J. et al., 2022). BSFL can convert organic waste with low nutritional value into high-quality protein biomass (Seyedalmoosavi et al., 2022). Edible insect BSFL is a quality protein resource that can be used as a feed protein supplement for fisheries, poultry. It is of great significance for alleviating animal feed protein shortage (Cardinaletti et al., 2022; Lu, S. et al., 2022; Cheng et al., 2023). The addition of microbes can improve the organic waste conversion efficiency of BSFL (Zhang et al., 2021). BSFL gut microbiota expedite the bioconversion of organic waste and facilitate nutrient conversion from waste (Jiang et al., 2019; Ao et al., 2021). Gut microbes therefore play an important role in the BSFL process of waste conversion. BSFL has higher feed conversion efficiency than yellow mealworms and house crickets (Oonincx et al., 2015). But the role played by BSFL gut microbes in this process is currently unknown.

Insects acquire protein from food, and the first is to degrade the protein. Protein degradation by proteases occurs mainly in the insect midgut (Chapman et al., 2014). Serine proteases include trypsin, chymotrypsins, and elastases. Serine proteases account for the majority of midgut protease activity in Dipterans (Holtof et al., 2019). Trypsin and chymotrypsin as major proteases in *Locusta migratoria*, in addition to a minor contribution of cysteine proteases to the total proteolytic activity in the gut (Spit et al., 2014). Insect gut proteases are produced by themselves or by gut microbes (Kannan et al., 2019). However, whether gut microbes promote protease production in insect hosts is currently unknown.

Different types of organic waste substrates are consumed by BSFL, and these produce fluctuations in the composition of the gut microbiota (De Smet et al., 2018). *Firmicutes* spp. are important in BSFL digestion of cattle and swine manure. Bacteroidetes and Proteobacteria, respectively, are the most abundant microbiota involved in the digestion of poultry manure and food waste (Zhan et al., 2020). An increase in the amount of protein in BSFL diet can increase the abundance of Proteobacteria and *Firmicutes*. However, the abundance of Bacteroidetes decreased with increasing protein content in the BSFL diet (Bruno et al., 2019). Different types of organic waste contain different nutrients for BSFL (Liu et al., 2022). These differences may cause changes in the gut microbial community.

BSFL gut microbes can help BSFL digest organic waste (Gold et al., 2018). However, few studies have focused on the effect of manure digestion on BSFL gut microbes (Liu et al., 2022). *Dysgonomonas*, *Morganella*, *Enterococcus*, *Pseudomonas*, *Actinomyces*, and *Providencia* were the predominant gut microbes identified when BSFL was fed on four types of organic waste (brewers’ spent grains, kitchen food waste, poultry manure, and rabbit manure; Tanga et al., 2021). BSFL gut microbes such as *Enterococcus*, *Cellulomonas*, *Pichia* yeasts, or filamentous *Fusarium* spp. increased BSFL degradation of lignocelluloses in palm kernel meal (Klüber et al., 2022). *Enterococcus*, *Providencia*, and *Morganella* were the dominant genera when BSFL was reared in swine or chicken manure (Ao et al., 2021). These studies focused on the changes of gut microbes during BSFL digestion of organic waste, but did not correlate the gut microbiota with digestion of specific nutrients.

BSFL has been used to degrade organic waste. Most of the omics data used to study the changes of gut microbiota composition involve 16S rRNA or 16S rDNA (Jiang et al., 2019; Wynants et al., 2019; Zhan et al., 2020; Pei et al., 2022). Few studies have focused on ITS1 sequence changes. More information on the composition and changes of gut microbiota in BSFL will increase understanding of how gut microbiota assist BSFL to degrade organic waste.

In this study, germ free BSFL and gnotobiotic BSFL were fed with high protein artificial medium. The microbiome of gnotobiotic BSFL (16S rDNA sequences and ITS1 sequences) gut was detected at three time points (0 day, 12 days, 24 days). In order to reveal the change pattern of gut microbiota in the artificial feed digested by BSFL and better explain the reason why BSFL efficiently degrades protein with the help of its gut microbiota.

## 2. Materials and methods

### 2.1. Black soldier fly source

Black soldier fly larvae were obtained from Huazhong Agricultural University, Wuhan, China. The Wuhan strain was established from eggs collected at a poultry facility in the Wuhan suburbs in November 2008 (Cai et al., 2018). Colonies were maintained on a standard diet (Wheat bran: wheat middlings =1:1; Sheppard et al., 2002).

### 2.2. Dissection of BSFL gut samples

We dissected BSFL gut samples on a UV-sterilized ultra-clean bench. The BSFL were removed from the artificial diet with autoclave-sterilized tweezers and rinsed with autoclave-sterilized water. Then, the BSFL was immersed in 75% ethanol for 2–3 min to surface sterilize them. After that, the BSFL surface was rinsed again with autoclaved sterile water. The BSFL gut was accessed using a sterilized dissection needle and placed in a sterilized 5-ml internal rotation cryopreservation tube. We froze the samples in liquid nitrogen for 15 min, and stored them at −80°C.

### 2.3. Preparation of germ-free BSFL (GF) and gnotobiotic BSFL (GB)

We collected about 5 g of fresh BSF eggs produced within 6 h and put them into autoclave-sterilized water. The eggs were dispersed with a soft brush, and autoclaved filter paper separated the eggs from water. Then, the eggs were transferred to a 15-mL autoclave-sterilized centrifuge tube, and added 10 ml Sporgon^®^ (Decon Labs, Inc. Bryn Mawr, PA, United States). The sterilized eggs were poured into a filter paper, followed by three washes with sterile water. The drained eggs were transferred to a sterile standard diet (sealed with 8 layers of sterile gauze), and placed in a 28°C incubator.

Guts from GF BSFL (after rearing for 15 days at 28°C) were dissected to verify their GF status. Gut homogenates were diluted 10^−2^ fold with sterile water. The homogenates were then spread on the surface of Nutrient Agar-NA (Solarbio^®^, N8290) or Potato Dextrose Agar-PDA (Solarbio^®^, P8931). The plates were incubated upside down for 48 h at 28°C. The E.Z.N.A.^®^ soil DNA kit (Omega Bio-Tek, D5625) was used to extract the DNA from gut homogenates.

We collected 10-day-old BSFL from standard feed and then surface sterilized using 75% ethanol for 2–3 min, followed by three washes with sterile water. Then, we collected 5 BSFL guts for homogenization. Sterile water was added in gut homogenization to 5 ml, then vortexed them with shaking for further use. GB BSFL was prepared by culturing 15-day-old GF BSFL and 1 ml of gut homogenates of 5 natural 10-day-old BSFL (fed on standard diet) together in artificial diet.

We tested whether GF BSFL remained germ-free during conversion and GB BSFL inoculated with the gut microbes. At the end of conversion, DNA was extracted from GF BSFL gut samples and GB BSFL gut samples. PCR verification was performed on 16S and ITS1. The primers used are shown in Table 1.

**Table 1:** PCR primers for germ-free BSFL (GF) and gnotobiotic BSFL (GB) validation.

Target genes	Primer sequences (5` → 3`)	Reference
16S rDNA	27F (5`-GTTTGATCCTGGCTCAG-3`)	Ceja-Navarro et al. (2015)
	1492R (5`-GGTTACCTTGTTACGACTT-3`)	
ITS1	ITS1 (5`-TCCGTAGGTGAACCTGCGG-3`)	White et al. (1990) and Gardes and Bruns (1993)
	ITS4 (5`-TCCTCCGCTTATTGATATGC-3`)	
*Tubulin*	F (5`-GCTCTCTACGACATCTGCTTTA-3`)	Gao et al. (2019)
	R (5`-CAGGTGGTAACTCCAGACATT-3`)	

### 2.4. Preparation of artificial diets

The artificial diet formula used was taken from the *Drosophila melanogaster* artificial diet (Piper et al., 2014), with some modifications. See Supplementary Tables S1, S2 for the formula of the artificial diet used. For the sterilization method of the artificial diet, see Supplementary Table S3. The reagents used for the artificial diets are shown in Supplementary Table S4.

### 2.5. Design of the experiment

We used germ-free BSFL (GF) and gnotobiotic BSFL (GB) to digest a high-protein artificial feed model. The experimental groups tested are shown in Table 2. Each group was prepared in triplicate.

**Table 2:** Design of the experiment.

Group	Experimental group setting	Artificial diet/g (dry matter)
GF	50 germ-free BSFL	50
GB	50 germ-free BSFL**+**1-mL gut homogenates of 5 natural 10-d-old BSFL	

Each group had three replications.

### 2.6. Determination of bio-physiological indicators

We examined the protein content of the artificial diets referencing the standards of the ministry of agriculture and rural affairs of the People’s Republic of China NY/T 3493–2019. The artificial diet was placed in an electric thermostatic drying oven at 60°C and oven dried to a constant weight. Then, the artificial diet protein reduction rate was determined as follows:


$$\mathrm{Artificial diet protein reduction rate}\;\left(\%\right)=\frac{\left(W1-W2\right)}{W1}\times 100.$$

W1 and W2 are the dry weight protein amounts of BSFL artificial diet before and after rearing, respectively.

Three kits were used to detect protein digestive enzyme activities of the of the GF and GB group gut samples. A trypsin activity assay kit (Solarbio^®^, BC2310) was used for trypsin and a pepsin activity assay kit (Solarbio^®^, BC2320) was used for pepsin. A leucine aminopeptidase (LAP) activity assay kit (Solarbio^®^, BC4140) was used for peptidases. Trypsin activity and pepsin activity were measured by UV spectrophotometry, and peptidase activity was measured by visible spectrophotometry. Enzyme activities were calculated according to sample protein concentrations. The detection methods used followed kit instructions.

### 2.7. 16S rDNA and its gene amplification and sequencing

We collected separate gut samples from the GF and GB groups. DNA was extracted from the samples using the E.Z.N.A.^®^ Stool Kit (Omega Bio-Tek, Norcross, GA, United States) following manufacturer instructions. The primers 806R (5`-GGACTACHVGGGTWTCTAAT-3`) and 338F (5`-ACTCCTACGGGAGGCAGCAG-3`) were used to amplify the bacterial V3–V4 region of 16S rDNA genes (Liu et al., 2016). The primers ITS1 (5`-CTTGGTCATTTAGAGGAAGTAA-3`) and ITS2 (5`-TGCGTTCTTCATCGATGC-3`) were used to amplify the fungus ITS1-ITS2 region of ITS1 genes (Wang et al., 2018). Purified PCR products were analyzed using the Illumina Miseq PE300 sequencing platform (Illumina, San Diego, CA, United States).

### 2.8. Data analysis

We processed the raw data using the standard procedures of Beijing Allwegene Technology Co., Ltd. The 16S rDNA and ITS gene sequences were processed and demultiplexed through QIIME2 (Version 1.8.0). Quality filtering and trimming, denoising, pair-end read merging, chimeric removal and taxonomy assignments were processed by Vsearch (Version 2.7.1). Analyses were performed using IBM SPSS 20.0 (IBM, Chicago, IL, United States).

Clean tags were used to cluster gene sequences (or reduce noise) to generate operational taxonomic units (OTUs). The clustering method could be selected as uparse, uclust, or ref. reference database, with the default being uparse. The noise reduction method used was the unoise3 method (Edgar, 2010, 2013; Rognes et al., 2016).

### 2.9. Statistical analysis

We performed three replications for all experiments. Data are presented as the mean ± standard error (SE). We analyzed the results of bio-physiological indicators using one-way analysis of variance (ANOVA) and Tukey’s *post hoc* comparisons. Student’s *t*-test was used to analyze the results of artificial diet protein reduction rate. The number of replicate samples for all analyses was three. Shannon rarefaction curves and other richness and diversity indices of bacterial community (Chao1, observed_species and PD_whole_tree) were estimated using the QIIME2 platform. SPSS 20.0 (IBM SPSS Inc., Chicago, IL, United States). The free online platform of Allwegene cloud V2.0 Platform^1^ and a free online platform^2^ were used to analyze the statistical differences. Spearman’s rank correlation analysis was used to determine the significant correlation among the relative abundance of dominant microbes. *p <* 0.05 was considered statistically significant.

### 2.10. Network analysis

We used amplicon (16S rDNA and ITS1) abundances top20 (Supplementary Table S13) to calculate the correlation coefficient among the gut microbes. Co-occurrence network analysis was conducted at the genus level for the top 20 abundances of all samples (16S and ITS).

We used 16S rDNA amplicon OTU abundance (Supplementary Table S14) and 16S rDNA amplicon KEGG enrichment abundance (Supplementary Table S15) to calculate the 16S rDNA amplicon correlation coefficient between GB BSFL gut microbes and KEGG enrichment abundance. For network analysis of the co-occurrence patterns between protein metabolism and 16S rDNA amplicon (genus level) during BSFL conversion to artificial diets.

We used mixed data (16S rDNA amplicon OTU abundance Supplementary Table S14 and ITS1 amplicon OTU abundance Supplementary Table S16) and GB BSFL gut samples’ proteolytic enzyme activity (Supplementary Table S5) to calculate correlation coefficient between GB BSFL gut microbes and protein digestive enzymes. For the network analysis among 16S rDNA amplicon (genus level) abundance, ITS1 amplicon (genus level) abundance, and protein digestive enzyme activity data.

We used the Spearman test method on the calculated results to filter out the *p* values greater than 0.05 and values of R less than 0.6 (*R* > 0.6, *p* < 0.05; de Vries et al., 2018).

The 16S rDNA amplicon was used to discriminate bacteria, and the ITS1 amplicon was used to discriminate fungi. Calculation of the Spearman correlation coefficient was performed on the Allwegene cloud V2.0 Platform (see Footnote 1).

### 2.11. Data visualization

A Venn diagram and flower diagram were plotted by the online platform.^4^ Cytoscape (v3.9.1) was used for network analysis data visual plotting. GraphPad Prism 9 was used for other data visualization plots.

## 3. Results

### 3.1. Comparison of protein degradation and gut protein digestion enzyme activity between the GF and GB BSFL

**Figure 1:**
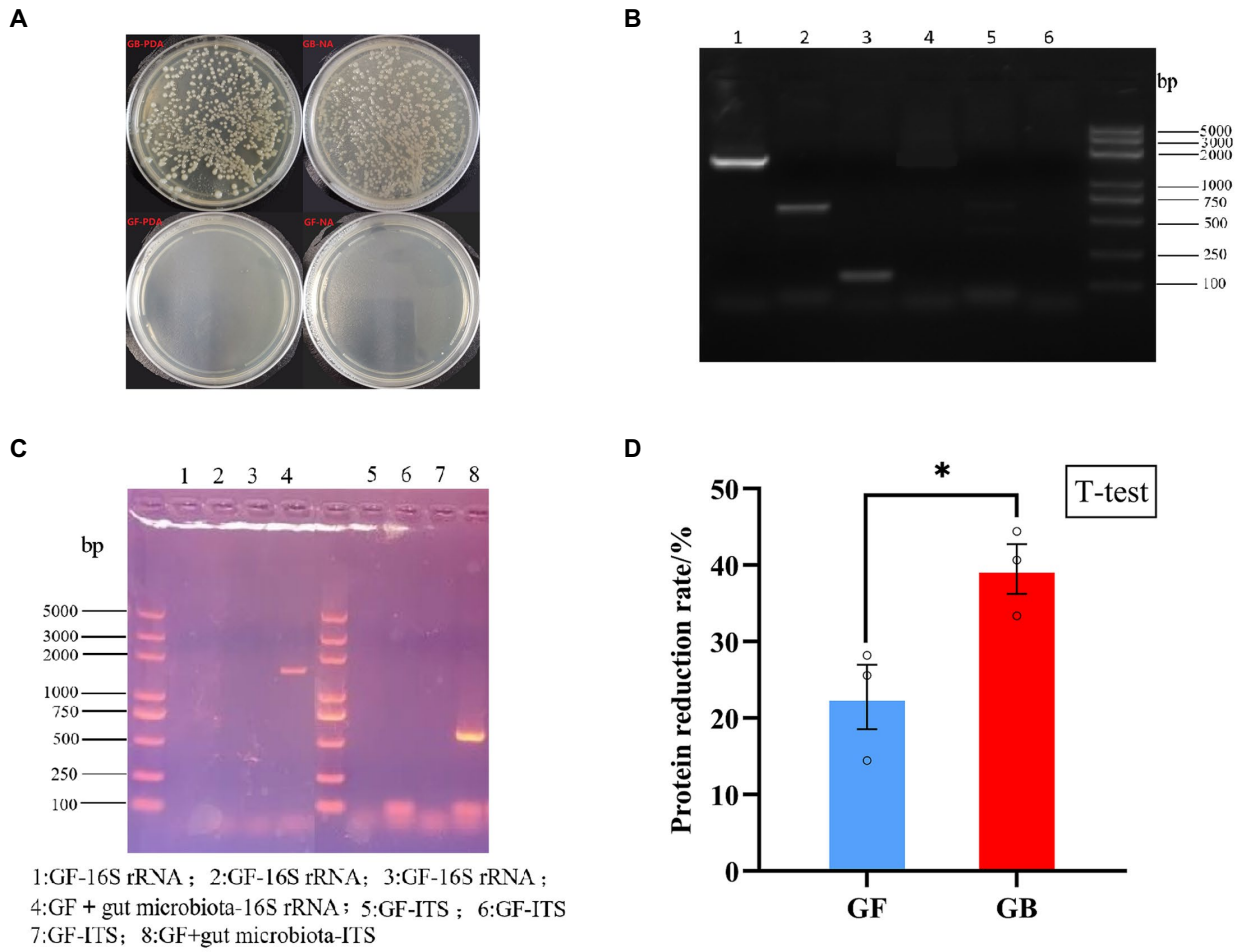
Validation of germ free BSFL and gnotobiotic BSFL and artificial diet protein reduction rate, **(A)** Germ free BSFL Plate validation, GF-germ-free BSFL,GB-gnotobiotic BSFL; **(B)** Germ free BSFL polymerase chain reaction (PCR) validation, lane1: gnotobiotic BSFL-16S-rDNA; lane2: gnotobiotic BSFL-ITS; lane3: gnotobiotic BSFL –Tubulin; lane4: Germ free BSFL-16S-rDNA; lane 5: Germ free BSFL-ITS; lane6: Germ free BSFL –Tubulin; **(C)** Germ free BSFL and gnotobiotic BSFL polymerase chain reaction (PCR) validation after the end of conversion; **(D)** GF-germ-free BSFL; GB-gnotobiotic BSFL, *p ≤ 0.05.

The validation results of GF and GB BSFL are shown in Figures 1A–C. To evaluate protein degradation efficiency of the GF and GB groups, we calculated the protein reduction rate of dry artificial diet after conversion by GF and GB BSFL. Then, the protein reduction rate of the food dry matter was calculated. There was a significant difference in the protein reduction rate between GF and GB artificial food residues (Figure 1D). The protein reduction rate of the GB artificial feed residues was significantly higher than that of the GF artificial food residues. The protein reduction rate of GB increased by 73.44% compared to the GF group (Table 3).

**Table 3:** Artificial feed protein degradation rate.

Group	Protein degradation rate/% (Mean ± SE)
GF	22.78 ± 4.21
GB	39.51 ± 3.24

**Figure 2:**
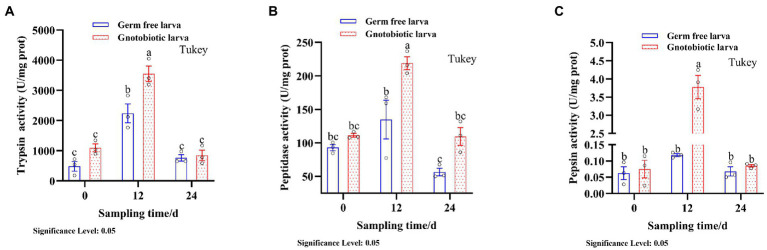
Protein digestive enzyme activities of gut samples, **(A)** trypsin activity; **(B)** peptidase activity; **(C)** pepsin activity. The group with the largest mean is annotated with the letter a. If there are statistically significant differences between groups, different letters shall be marked; otherwise, the same letters shall be marked.

The presence of gut microbiota effected BSFL gut protein digestive capacity. This effect was manifested by changes in the activities of gut protein digestive enzymes. To evaluate this, protein digestive enzyme activities of GF and GB gut samples were determined (Figure 2). At 12 days, the activities of protein digestive enzymes were significantly different between GF and GB. However, the protein digestive enzyme activities of GF and GB were not significant at 0 day and 24 days, and GF was slightly lower than GB. During the whole process, trypsin activity was the highest, followed by peptidase activity and pepsin activity was the lowest (Table 4). During the whole process, the protein digestion enzyme activities of GF and GB both showed an initially increasing and then a decreasing trend. These results suggest that the enhanced gut protein degradation ability of GB BSFL resulted from the enhanced activities of protein digestive enzymes associated with the presence of gut microbes. Trypsin and peptidase play a major role in protein degradation by BSFL.

**Table 4:** Gut proteolytic enzyme activity during BSFL conversion to artificial diets.

Group	Trypsin activity (U/mg prot)	Peptidase activity (U/mg prot)	Pepsin activity (U/mg prot)
GB-0d	1097.765 ± 132.019c	111.278 ± 2.792bc	0.075 ± 0.027b
GB-12d	3544.028 ± 256.285a	219.009 ± 9.601a	3.776 ± 0.321a
GB-24d	850.851 ± 173.165c	109.670 ± 13.243bc	0.085 ± 0.004b
GF-0d	484.505 ± 160.235c	92.929 ± 4.598bc	0.062 ± 0.020b
GF-12d	2240.317 ± 311.964b	134.824 ± 28.828b	0.118 ± 0.005b
GF-24d	764.226 ± 109.175c	56.379 ± 5.650c	0.068 ± 0.014b

The group with the largest mean is annotated with the letter a. If there are statistically significant differences between groups, different letters shall be marked; otherwise, the same letters shall be marked.

### 3.2. Analysis of the BSFL gut microorganisms

In order to reveal the change pattern of gut microbiota in the artificial feed converted by BSFL and better explain the reason why BSFL efficiently degrades protein with the help of its gut microbiota. The microbiome (16S rDNA and ITS1) of gnotobiotic BSFL gut was detected at three time points (day 0, day 12, and day 24).

A total of 190 OTUs were generated from the ITS1 sequences and 133 OTUs were generated from the 16S rDNA sequences. Statistics of OTU numbers for individual samples are shown in Table 5.

**Table 5:** Statistics of OTU numbers for individual samples.

SampleID	OTUs	SampleID	OTUs
GB0dITS1_1	57	GB0d16S_1	98
GB0dITS1_2	49	GB0d16S_2	104
GB0dITS1_3	44	GB0d16S_3	101
GB12dITS1_1	38	GB12d16S_1	60
GB12dITS1_2	35	GB12d16S_2	64
GB12dITS1_3	13	GB12d16S_3	53
GB24dITS1_1	123	GB24d16S_1	72
GB24dITS1_2	60	GB24d16S_2	57
GB24dITS1_3	18	GB24d16S_3	64

#### 3.2.1. BSFL gut amplicon α diversity index and β diversity index

GB gut sample amplicons 16S rDNA sequence of α diversity index (Supplementary Figure S1) decreased with time. However, the ITS1 sequence of α diversity index (Supplementary Figure S2) increased with time. The trends of α diversity between 16S rDNA sequence and ITS1 sequence were different. These results indicate that the times that bacteria and fungi played roles in the conversion process differed.

GB gut samples amplicons 16S rDNA sequence of β diversity index (Supplementary Figures S3A–C) were similar (*p* > 0.05). However, the ITS1 sequence of the β diversity index (Supplementary Figures S3D–F) showed a strongly significant difference (*p* < 0.01). The variation trend of β diversity between 16S rDNA sequence and ITS1 sequence was different. These results suggest that the abundance of fungi varied significantly during the conversion process.

#### 3.2.2. Changes in gut microbial abundance

We initially defined the gut microbes present in all GB BSFL gut samples as the core gut microbes. The Venn diagram (Supplementary Figure S4) of gut microbiota amplicons showed that 16S rDNA sequences (Supplementary Figures S4A–C) at the OTU, genus, and species levels of the core microbes accounted for total amounts of 43.6, 52.0, and 47.6%, respectively. A list of 16S rDNA sequences for core microbes is given in Supplementary Table S6. ITS1 sequences (Supplementary Figures S4D–F) at the OTU, genus, and species levels of core microbes accounted for total amounts of 15.8, 26.9, and 21.2%, respectively. The detailed list of ITS1 sequences for core microbes is presented in Supplementary Table S7.

The flower diagram (Supplementary Figure S5) of all samples indicated that there were 24 core OTUs for the 16S rDNA sequence (Supplementary Figure S5A) and 4 core OTUs for ITS1 sequence (Supplementary Figure S5B). Detailed lists of these OTUs are shown in Tables 6, 7. These core OTUs may be important in the conversion of artificial diets by BSFL.

**Table 6:** All gnotobiotic samples core OTUs for 16S rDNA amplicons.

OTUs	Level	Taxonomy
OTU_10	species	*s__Heliconius_timareta_timareta*
OTU_108	species	*s__uncultured_Actinomycetales_bacterium*
OTU_11	species	*s__Proteus_mirabilis_WGLW4*
OTU_115	species	*s__bacterium_NLAE-zl-C91*
OTU_118	genus	*g__Campylobacter*
OTU_12	species	*s__Klebsiella_pneumoniae_BIDMC_21*
OTU_121	family	*f__Lachnospiraceae*
OTU_14	species	*s__Lactobacillus_plantarum*
OTU_15	species	*s__Escherichia_coli*
OTU_16	species	*s__Lactobacillus_brevis*
OTU_19	species	*s__Cronobacter_dublinensis_subsp._lactaridi_LMG_23825*
OTU_2	species	*s__human_gut_metagenome*
OTU_21	family	*f__Orbaceae*
OTU_22	species	*s__Enterococcus_ureilyticus*
OTU_3	species	*s__Enterococcus_moraviensis_ATCC_BAA-383*
OTU_35	species	*s__Enterococcus_termitis*
OTU_4	species	*s__Morganella_morganii*
OTU_6	genus	*g__Vagococcus*
OTU_76	species	*s__Heliconius_numata_bicoloratus*
OTU_77	genus	*g__Dysgonomonas*
OTU_78	genus	*g__Dysgonomonas*
OTU_8	family	*f__Orbaceae*
OTU_81	species	*s__Heliconius_numata_bicoloratus*
OTU_9	species	*s__Exiguobacterium_mexicanum*

**Table 7:** All gnotobiotic samples core OTUs for ITS1 amplicons.

OTUs	Level	Taxonomy
OTU_127	species	*s__Issatchenkia_orientalis*
OTU_144	order	*o__Saccharomycetales*
OTU_153	species	*s__Candida_tropicalis*
OTU_33	species	*s__Issatchenkia_orientalis*

**Figure 3:**
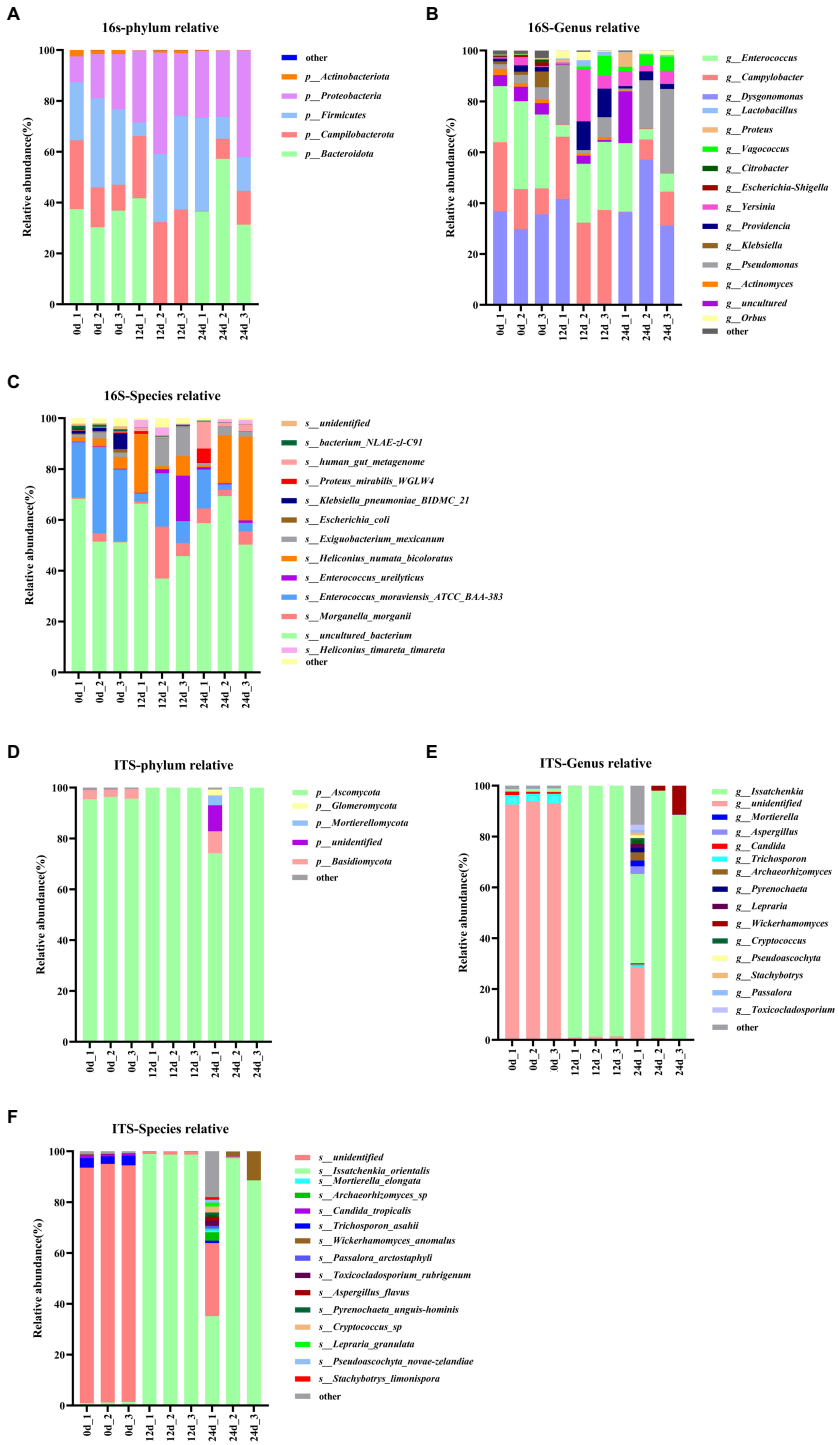
Relative abundance changes of core gut microbes, 16S rDNA sequences: **(A)** Phylum level, **(B)** genus level, **(C)** species level; ITS1 sequences: **(D)** phylum level, **(E)** genus level, **(F)** species level.

In this study, BSFL was fed with artificial diet (approximately 47.5% protein; protein/carbohydrate ratio, 1:1). The 16S rDNA genes showing the highest relative abundance in the gut microbiota were Proteobacteria, *Firmicutes*, and Bacteroidotain (Figure 3A; Table 8 16S-Relative abundance). Ascomycota was the ITS1 gene showing the highest relative abundance (Figure 3D). During the 0- to 12-day of conversion, the *Firmicutes* of the BSFL gut microbiota were enriched while the Bacteroidetes were reduced. This situation was reversed during the 12 -to 24-day period of conversion (Supplementary Figure S6). The Ascomycota of the BSFL gut microbiota was enriched throughout the conversion, which was dominated by *Issatchenkia* spp. (Supplementary Figure S7). These results suggest that, in addition to the *Firmicutes*, *Issatchenkia* spp. belonging to the Ascomycota may also be important during protein degradation.

**Table 8:** Relative abundance of amplicons.

Sample_ID	GB0d_1	GB0d_2	GB0d_3	GB12d_1	GB12d_2	GB12d_3	GB24d_1	GB24d_2	GB24d_3	Classification
p__Bacteroidota	37.40	30.28	36.87	41.70	0.13	0.50	36.48	57.21	31.33	16S-Phylum
p__Campilobacterota	27.05	15.66	10.18	24.45	32.22	36.80	0.19	7.85	13.24	
p__*Firmicutes*	22.90	35.07	29.75	5.34	26.73	36.70	36.59	8.66	13.29	
p__Proteobacteria	10.19	17.48	21.64	28.36	39.92	24.78	26.35	26.12	42.03	
p__Actinobacteriota	2.46	1.51	1.56	0.14	1.00	1.22	0.37	0.15	0.11	
others	0.00	0.00	0.00	0.00	0.00	0.00	0.02	0.00	0.00	
g__*Dysgonomonas*	36.92	29.88	35.67	41.70	0.13	0.49	36.48	57.21	31.33	16S-Genus
g__*Campylobacter*	27.05	15.66	10.18	24.45	32.22	36.80	0.19	7.85	13.24	
g__*Enterococcus*	22.07	34.53	28.99	4.72	23.12	26.84	26.95	4.11	7.11	
g__uncultured	4.32	5.73	4.50	0.12	3.24	0.71	20.33	0.06	0.10	
g__*Actinomyces*	2.44	1.45	1.55	0.14	1.00	1.22	0.37	0.15	0.11	
g__*Pseudomonas*	1.81	3.20	4.65	23.15	1.14	7.72	0.74	18.92	32.95	
g__*Klebsiella*	1.11	1.22	6.20	0.05	0.02	0.03	0.04	0.02	0.05	
g__*Providencia*	1.10	2.50	1.81	0.11	11.28	11.23	0.93	3.48	2.00	
g__Yersinia	0.55	3.25	0.34	0.89	20.44	5.17	5.82	2.41	5.15	
g__*Escherichia*-*Shigella*	0.41	0.54	1.45	0.01	0.01	0.00	0.01	0.00	0.01	
g__Citrobacter	0.28	0.36	1.08	0.01	0.02	0.00	0.01	0.00	0.01	
g__*Vagococcus*	0.15	0.15	0.22	0.05	1.12	7.74	1.78	4.25	5.61	
g__*Proteus*	0.09	0.09	0.34	0.99	0.05	0.02	5.58	0.01	0.06	
g__*Lactobacillus*	0.04	0.04	0.04	0.53	2.43	1.58	0.30	0.30	0.55	
g__*Orbus*	0.02	0.04	0.05	3.01	3.26	0.39	0.04	1.18	1.66	
others	1.65	1.36	2.94	0.06	0.51	0.06	0.45	0.05	0.08	
s__uncultured_bacterium	68.30	51.44	51.16	66.40	36.92	45.77	58.70	69.38	50.31	16S-Species
s__*Morganella_morganii*	0.55	3.25	0.34	0.89	20.44	5.17	5.82	2.41	5.15	
s__*Enterococcus_moraviensis*_ATCC_BAA-383	21.71	33.99	28.35	3.18	20.99	8.57	15.28	2.16	3.37	
s__*Enterococcus_ureilyticus*	0.16	0.21	0.26	0.19	1.60	17.87	0.96	0.43	0.99	
s__*Heliconius_numata*_bicoloratus	1.80	3.18	4.62	23.11	1.14	7.71	0.72	18.90	32.94	
s__*Exiguobacterium_mexicanum*	1.05	2.38	1.74	0.10	11.12	11.22	0.92	3.44	1.98	
s__*Escherichia_coli*	0.38	0.52	1.38	0.01	0.01	0.00	0.01	0.00	0.01	
s__*Klebsiella_pneumoniae*_BIDMC_21	1.11	1.22	6.20	0.05	0.02	0.03	0.04	0.02	0.05	
s__*Proteus_mirabilis*_WGLW4	0.09	0.09	0.34	0.99	0.05	0.02	5.58	0.01	0.06		
s__human_gut_metagenome	0.16	0.31	0.34	1.33	0.48	0.39	10.70	1.51	2.72	
s__bacterium_NLAE-zl-C91	1.68	0.86	0.89	0.08	0.34	0.68	0.31	0.13	0.09	
s__unidentified	0.83	0.54	1.16	0.01	0.03	0.02	0.02	0.00	0.00	
s__*Heliconius_timareta*_timareta	0.02	0.04	0.05	3.00	3.19	0.39	0.04	1.16	1.63	
others	2.16	1.97	3.17	0.66	3.67	2.17	0.90	0.43	0.70	
p__Ascomycota	95.40	96.35	95.62	99.94	99.95	100.00	74.17	99.90	99.99	ITS1-Phylum
p__Basidiomycota	3.74	2.98	3.84	0.03	0.03	0.00	8.66	0.04	0.00	
p__unidentified	0.05	0.01	0.00	0.01	0.00	0.00	10.20	0.04	0.00	
p__Mortierellomycota	0.01	0.01	0.00	0.00	0.00	0.00	3.88	0.01	0.00	
p__Glomeromycota	0.00	0.00	0.00	0.00	0.00	0.00	2.32	0.00	0.00	
other	0.81	0.65	0.53	0.02	0.00	0.00	0.76	0.00	0.00	
g__unidentified	92.59	93.83	93.01	0.89	1.23	1.26	28.76	0.60	0.01	ITS1-Genus
g__Trichosporon	3.73	2.97	3.84	0.03	0.03	0.00	0.98	0.03	0.00	
g__*Candida*	1.35	0.82	0.67	0.02	0.00	0.02	0.43	0.11	0.02	
g__*Issatchenkia*	0.97	1.18	1.45	98.96	98.71	98.72	35.13	97.30	88.56	
g__*Aspergillus*	0.16	0.17	0.20	0.00	0.00	0.00	2.98	0.01	0.00	
g__*Mortierella*	0.01	0.01	0.00	0.00	0.00	0.00	2.28	0.01	0.00	
g__*Archaeorhizomyces*	0.01	0.00	0.00	0.00	0.00	0.00	3.26	0.01	0.01	
g__Pyrenochaeta	0.00	0.00	0.00	0.00	0.00	0.00	1.71	0.00	0.00	
g__Lepraria	0.00	0.00	0.00	0.00	0.00	0.00	1.53	0.00	0.00	
g__Wickerhamomyces	0.00	0.00	0.00	0.00	0.00	0.00	0.01	1.89	11.39	
g__*Cryptococcus*	0.00	0.00	0.00	0.00	0.00	0.00	2.32	0.00	0.00	
g__Pseudoascochyta	0.00	0.00	0.00	0.00	0.00	0.00	1.12	0.00	0.00	
g__*Stachybotrys*	0.00	0.00	0.00	0.00	0.00	0.00	1.03	0.01	0.00	
g__*Passalora*	0.00	0.00	0.00	0.00	0.00	0.00	1.01	0.00	0.00	
g__*Toxicocladosporium*	0.00	0.00	0.00	0.00	0.00	0.00	2.09	0.00	0.00	
others	1.18	1.01	0.83	0.09	0.02	0.00	15.36	0.02	0.01	
s__*Issatchenkia_orientalis*	0.97	1.18	1.45	98.96	98.71	98.72	35.13	97.30	88.56	ITS1-Species
s__unidentified	92.59	93.83	93.01	0.89	1.23	1.26	28.76	0.60	0.01	
s__*Trichosporon_asahii*	3.73	2.97	3.84	0.03	0.03	0.00	0.98	0.03	0.00	
s__*Candida_tropicalis*	1.35	0.82	0.67	0.02	0.00	0.02	0.00	0.11	0.02	
s__*Archaeorhizomyces*_sp	0.01	0.00	0.00	0.00	0.00	0.00	3.26	0.01	0.01	
s__*Mortierella_elongata*	0.00	0.01	0.00	0.00	0.00	0.00	1.50	0.00	0.00	
s__*Wickerhamomyces_anomalus*	0.00	0.00	0.00	0.00	0.00	0.00	0.01	1.89	11.39	
s__*Passalora_arctostaphyli*	0.00	0.00	0.00	0.00	0.00	0.00	1.01	0.00	0.00	
s__*Toxicocladosporium_rubrigenum*	0.00	0.00	0.00	0.00	0.00	0.00	2.09	0.00	0.00	
s__Aspergillus_flavus	0.14	0.16	0.20	0.00	0.00	0.00	1.50	0.00	0.00	
s__*Pyrenochaeta_unguis-hominis*	0.00	0.00	0.00	0.00	0.00	0.00	1.71	0.00	0.00	
s__*Cryptococcus*_sp	0.00	0.00	0.00	0.00	0.00	0.00	2.32	0.00	0.00	
s__*Lepraria_granulata*	0.00	0.00	0.00	0.00	0.00	0.00	1.53	0.00	0.00	
s__*Pseudoascochyta_novae-zelandiae*	0.00	0.00	0.00	0.00	0.00	0.00	1.12	0.00	0.00	
s__*Stachybotrys_limonispora*	0.00	0.00	0.00	0.00	0.00	0.00	1.03	0.01	0.00	
other	1.21	1.02	0.84	0.09	0.02	0.00	18.05	0.04	0.01	

The relative abundance changes of gut microbes are shown in Figure 3. ITS1 sequences relative abundance show that at the phylum level (Figure 3D) the relative abundance of Ascomycota in the full process of conversion was dominant (all above 85%). At the genus level (Figure 3E), the relative abundance of unidentified gut microbiota decreased from 93% to less than 1% during conversion. The relative abundance of *Issatchenkia* spp. increased sharply from 1.1 to 90%. A similar situation occurred at the species level (Figure 3F). The relative abundance of *Issatchenkia orientalis* increased from 1.2 to 98% during the conversion process (Table 8 ITS1-Relative abundance). This suggests that *Issatchenkia* spp. may play an important role in protein degradation and this role may be indirect.

### 3.3. Correlation analysis of the gut microbiota amplicons of the top 20 genera in abundance

We explored the interaction between the gut microbiota during the conversion process. The genus levels of the top 20 abundances of all samples were selected for correlation analysis (Supplementary Table S8). Correlation analysis data between16S rDNA bacteria and ITS1 fungi are presented in Supplementary Table S9.

**Figure 4:**
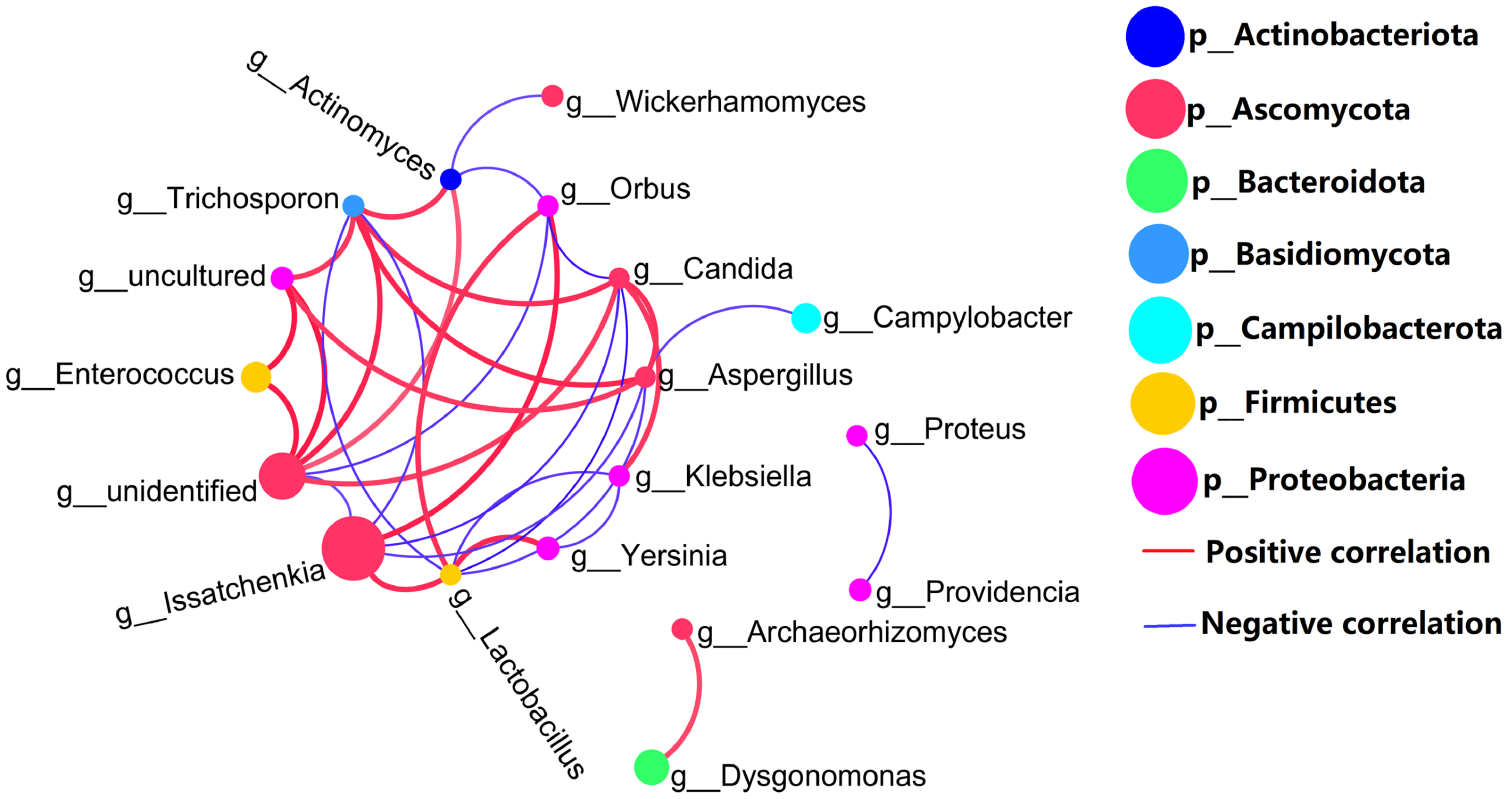
Genus levels of the top 20 abundances of all samples co-occurrence network, 16S rDNA sequence and ITS1 sequences, the average number of neighbors for this network is 4.429; characteristic path length is 1.956; network density is 0.341; and the clustering coefficient is 0.521.

Co-network analysis of 16S rDNA bacteria and ITS1 fungi is shown in Figure 4. Proteobacteria (bacteria) and Ascomycetes (fungi) are the most common microbial types in top 20 genera. Certainly, the presence of uncultured spp. (Proteobacteria) and unidentified spp. (Ascomycetes) in top 20 genera which may disturb the focus of the analysis. In addition to uncultured spp. (Proteobacteria) and unidentified spp. (Ascomycetes), the highest abundant *Issatchenkia* spp. and other gut microbes were mostly negatively correlated. But, fungal *Issatchenkia* spp. and bacterial *Lactobacillus* spp. and *Orbus* spp. had a positive correlation.

### 3.4. Prediction of gut microbiota function

**Figure 5:**
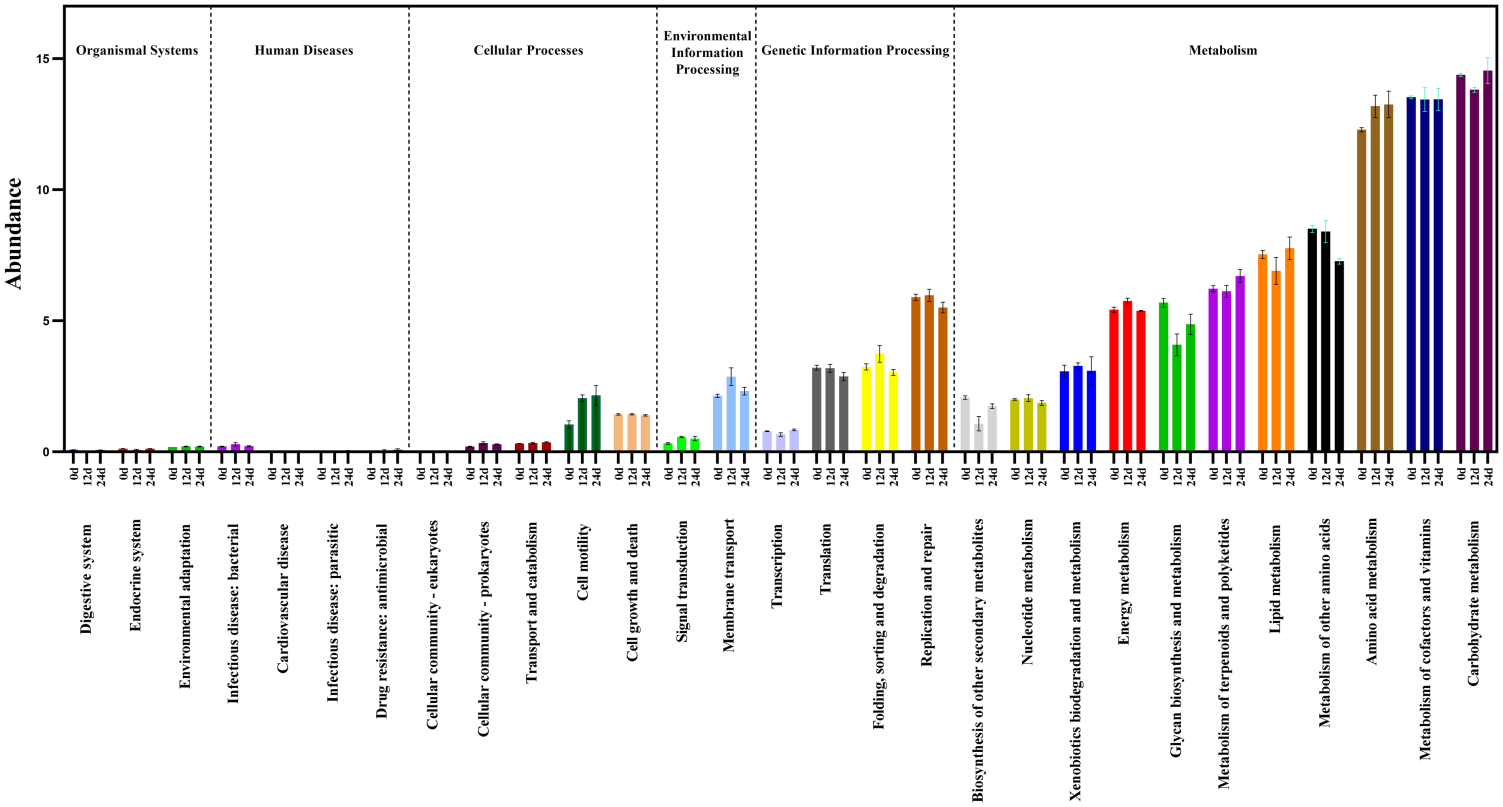
Prediction of 16S rDNA gene amplification functional pathways.

We used PICRUSt 2 (Douglas et al., 2020) to predict the 16S rDNA gene amplification function during BSFL conversion to artificial food. Functional pathways of the GB 16S rDNA gene (Figure 5) showed that carbohydrate metabolism, metabolism of cofactors and vitamins and amino acid metabolism were the top three in abundance of bacteria functional pathways. Adding the bacterial abundance of amino acid (proteinogenic amino acid) metabolism and metabolism of other amino acids (non-protein amino acids) exceeded that of the pathways of carbohydrate metabolism and cofactors and vitamins metabolism. These results show that amino acid and other amino acids metabolism are the most abundant of all functional pathways.

**Figure 6:**
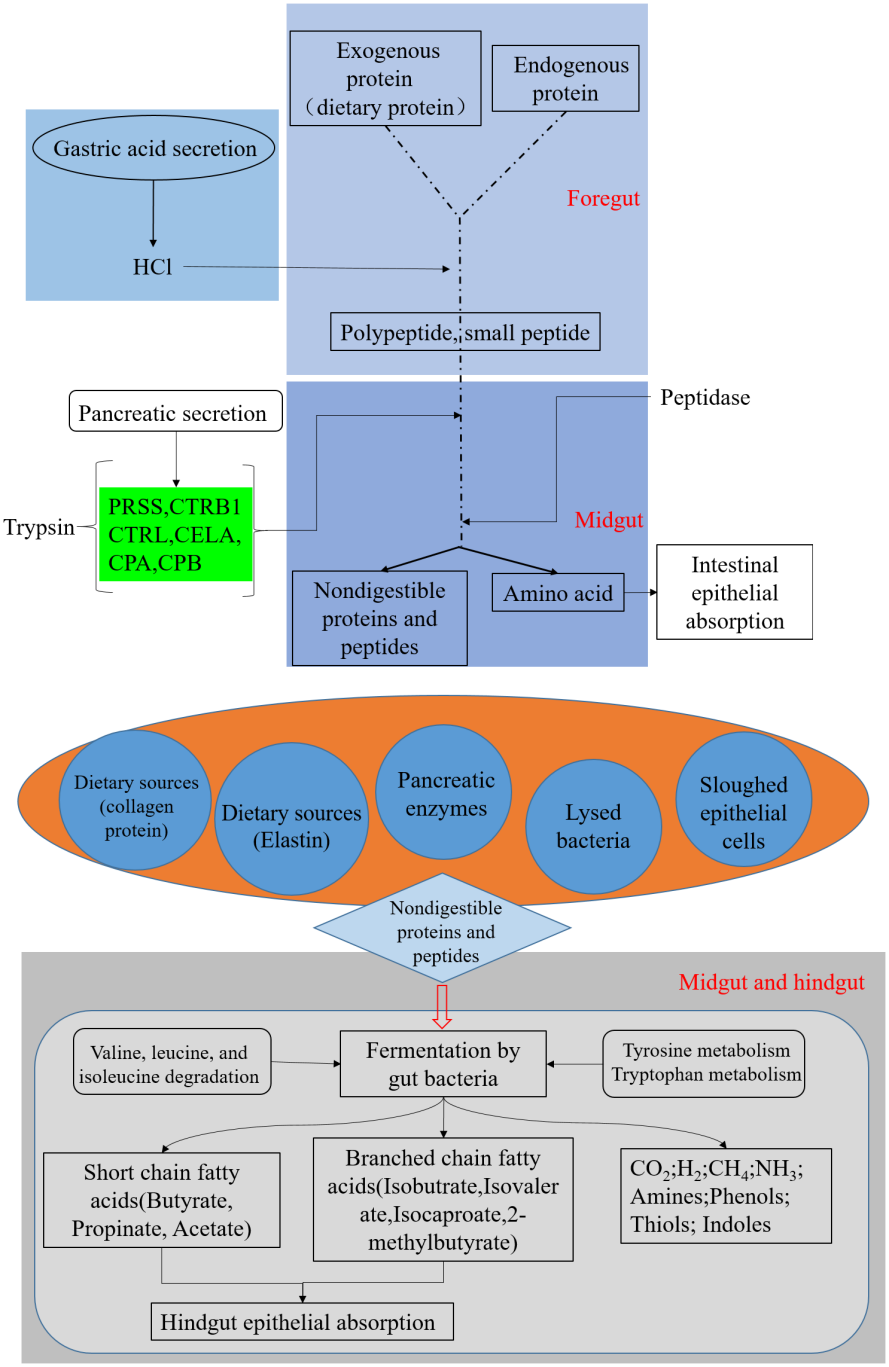
Black soldier fly genomic pathway annotation-protein digestion, reference to KEGG map04974, green boxes represent genes annotated on the genome.

Prediction of 16S rDNA gene amplification functional KEGG pathways (Figure 5) showed that gut microbiota abundance enriched by digestive system pathways was much lower than that enriched by pathways associated with amino acid metabolism. This suggests that in the presence of the BSFL host, protein degradation is dominated by the host itself. This is unlike the situation with microorganisms during composting. The main function of BSFL gut bacteria is amino acid metabolism, followed by protein degradation. We used online annotation tools^5^ for analysis of published genomic data on the black soldier fly (Zhan et al., 2020; Generalovic et al., 2021). We plotted black soldier fly pathways of protein digestion and absorption-map04974 (Figure 6), which provided evidence supporting this inference.

#### 3.4.1. Correlation analysis between gut microbiota and protein metabolic pathways

We further analyzed the metabolic pathways involved in protein metabolism of BSFL gut microbiota. Correlation analysis was carried out between the abundance of 16S amplicons at the OTU level and the abundance of 16S amplicons enriched in KEGG metabolic pathway during BSFL conversion into artificial food. Selected KEGG pathways related to protein metabolism, included amino acid (proteinogenic amino acid) metabolism, metabolism of other amino acids (non-protein amino acids) and the digestive system (Supplementary Table S10).

**Figure 7:**
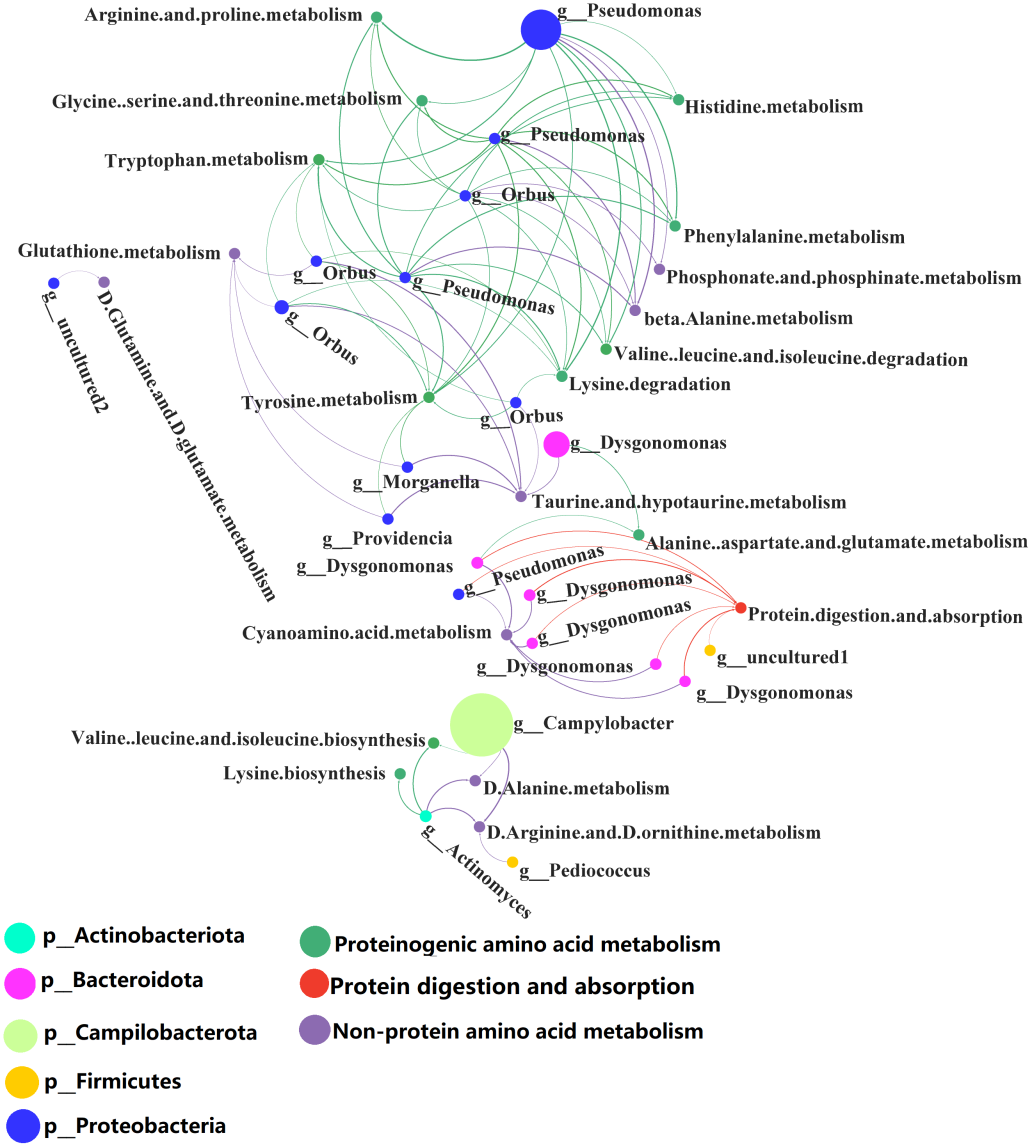
Network analysis of the co-occurrence patterns of protein metabolism and 16S rDNA amplicon (genus level) during conversion to artificial diets. Node size was proportional to node abundance; edge represents interactions between nodes. Sizes of connecting lines are based on r values. Nodes are colored for gut microbes, amino acid metabolism, metabolism of other amino acids and protein digestion and absorption, respectively. A connection was based on Spearman’s correlation coefficient (*R* > 0.6, *p* < 0.05). The average number of neighbors for this network is 4.625; characteristic path length is 4.159; network density is 0.149; and the clustering coefficient is 0.000.

Correlation analysis was performed between the selected metabolic pathways and the abundance of microbiota. Interactors with significant positive correlation (*R* > 0.6, *p* < 0.05) were selected (Supplementary Table S11) for the co-occurrence network analysis (Figure 7). The network contained 41 nodes with 83 edges.

Co-occurrence network analysis showed that the species and abundance of gut microbiota associated with proteinogenic amino acid metabolism and the metabolism of non-protein amino acids were higher than those associated with protein digestion and absorption. A detailed list of the protein metabolism involved by the BSFL gut bacteria is shown in Supplementary Tables S10, S11. *Pseudomonas* spp. were involved in proteinogenic amino acids metabolism. *Orbus* spp. and *Campylobacter* spp. were involved in the metabolism of non-protein amino acids. *Dysgonomonas* spp. were involved in protein digestion and absorption.

*Dysgonomonas* spp. were initially discovered as an opportunistic pathogen in humans, but they also occur in the gut of insects such as the honeybee, *Drosophila* and termites (Bridges and Gage, 2021). *Dysgonomonas* spp. has strong cellulose degrading ability and has been used to convert food waste (Xiong et al., 2019), lignocellulosic degradation and bioconversion of polysaccharides for biofuel production (Auer et al., 2017). Our research shows that *Dysgonomonas* spp. are involved in protein degradation and absorption in insect hosts. This extends the documented functions of *Dysgonomonas* spp.

Several *Pseudomonas* spp. have important applications in agriculture and biotechnology. In the present study, *Pseudomonas* spp. were involved in proteinogenic amino acids metabolism and this may assist the BSFL host in the synthesis of amino acids and proteins.

There are few reports about the biology of *Orbus* spp. Strains of this genus have been isolated from the gut of the butterfly *Sasakia charonda* (Kim et al., 2013). We found that *Orbus* spp. are strongly correlated with the metabolism of non-protein amino acids and are likely to be involved in the metabolism of non-protein amino acids processes with the host.

#### 3.4.2. Correlation analysis between gut microbiota and protein digestive enzyme activities

**Figure 8:**
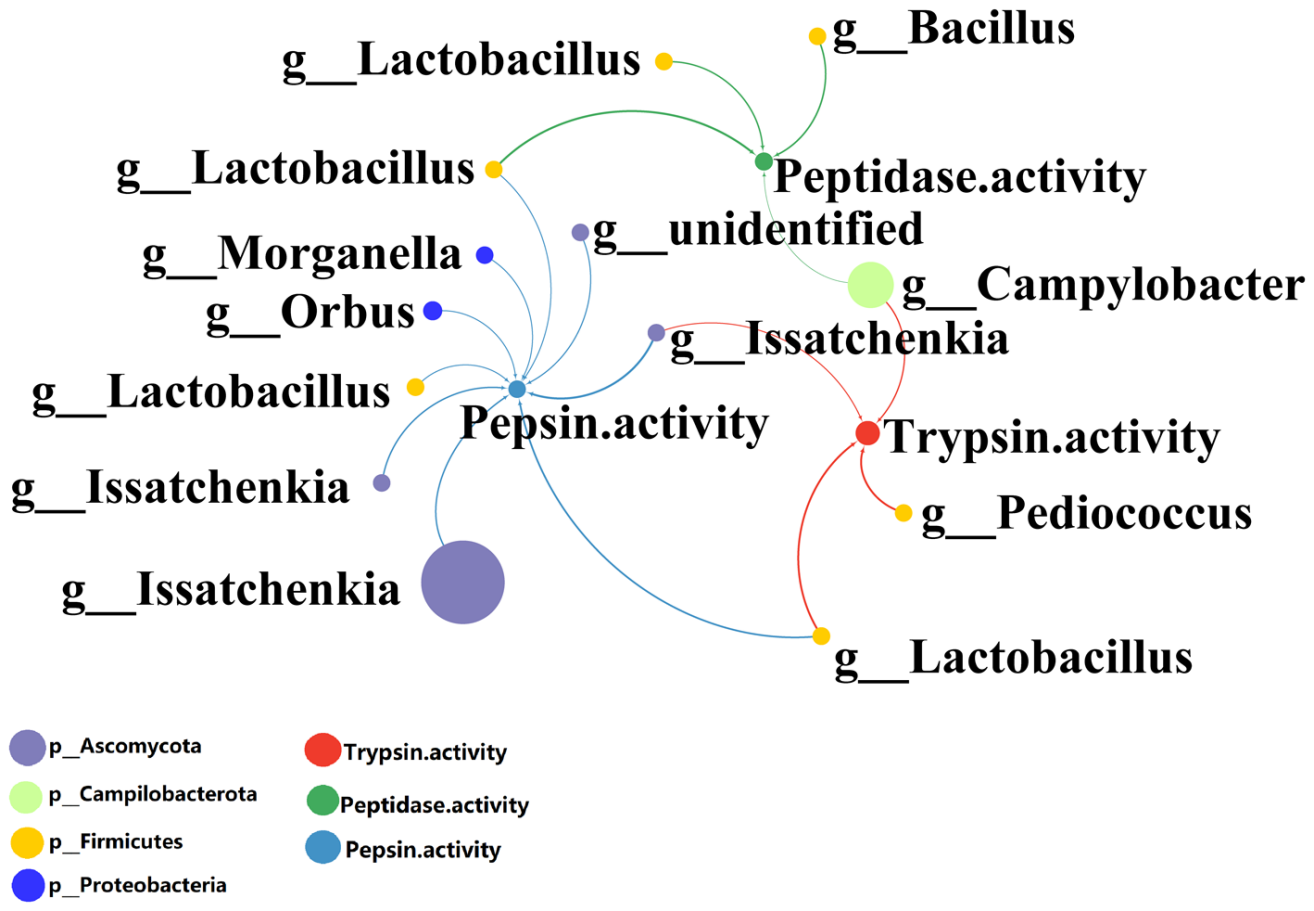
Network analysis of the co-occurrence patterns of protein digestion enzyme activity and 16S rDNA (genus level), ITS1 amplicon (genus level) during conversion to artificial diets. Node size was proportional to node abundance; edge represents interactions between nodes. Sizes of connecting lines are based on r values. Nodes were colored for bacteria, fungi, trypsin active, peptidase active, pepsin active, respectively. A connection was based on Spearman’s correlation coefficient (*R* > 0.6, *p* < 0.05). The average number of neighbors for this network is 2.125; characteristic path length is 2.588; network density is 0.142; and the clustering coefficient is 0.000.

The protein digestive enzyme activities of GB in BSFL guts were significantly higher than those of GF BSFL gut sample enzymes (Figure 2). We studied the link between the gut microbiota and protein digestive enzyme (trypsin, pepsin, peptidase) activities of the BSFL gut. Analysis of correlation between 16S rDNA amplicon (genus level) abundance, ITS1 amplicon (genus level) abundance, and protein digestive enzyme activity data was performed using Spearman’s coefficient. The interactors with a significant positive correlation (*R* > 0.6, *p* < 0.05) were selected (Supplementary Table S12) for co-occurrence network analysis (Figure 8). The network contains 16 nodes with 17 edges.

Correlation analysis between intestinal microbial abundance and proteolytic enzyme activity (Figure 8) showed that part of *Issatchenkia* spp. (Ascomycota) had the strongest correlation with pepsin activity. *Campylobacter* spp. (Campilobacterota), *Pediococcus* spp. (*Firmicutes*), and *Lactobacillus* spp. (*Firmicutes*) had the strongest correlation with trypsin activity. *Lactobacillus* spp. (*Firmicutes*) and *Bacillus* spp. (*Firmicutes*) had the strongest correlation to peptidase activity. Most of the gut microbes with a strong correlation with protein digestive enzyme activity were in the *Firmicutes*.

Some *Pediococcus* spp. and *Lactobacillus* spp. can produce specific proteases (Mustafa et al., 2020; Bansal et al., 2021). In this study, *Pediococcus* spp. and *Lactobacillus* spp. were found in the gut of BSFL and *Lactobacillus* spp., and *Pediococcus* spp. had a strong correlation with trypsin activity. These two genera may directly secrete proteolytic enzymes involved in protein degradation.

Some *Lactobacillus* spp., *Pediococcus* spp. and *Bacillus* spp. were significantly correlated with proteolytic enzymes (Supplementary Table S12) and some *Dysgonomonas* spp. were significantly correlated with protein digestion and absorption pathways (Supplementary Table S11). At the early stage (0–12 days) of BSFL conversion into artificial diets, *Lactobacillus* spp. were enriched while *Dysgonomonas* spp. were depleted (Supplementary Table S17 GB0d_to _GB12d; Supplementary Figure S6). This situation is contrary to that in the late conversion period (12 to 24 days) (Supplementary Table S17 GB12d_to _GB24d; Supplementary Figure S6). The *Issatchenkia* spp. of the BSFL gut microbiota presented enrichment throughout the conversion (Supplementary Table S18 GB0d_to _GB12d; Supplementary Table S18 GB0d_to _GB24d; Supplementary Figure S7). These results suggest that outside *Firmicutes*, *Issatchenkia* spp. may also play an important role in protein degradation. Taken together, these data suggest that there is a division of labor among BSFL gut core microbes in the catabolic metabolism of proteins.

## 4. Discussion

We found that the protein reduction rate of artificial diet after BSFL conversion significantly increased in the presence of gut microbiota. The proteome of the BSFL peripheral matrix revealed that BSFL gut protein digestion mainly relies on proteases. BSFL gut protein digestion enzymes mainly include trypsin and peptidase (Lin et al., 2021). Pepsin is rarely found in insect guts.

The majority of protease producing insect gut microbes are bacteria, including *Firmicutes*, Proteobacteria, and Actinobacteria. only one fungal member Ascomycota has been reported to produce proteases (Idowu et al., 2009; Banerjee et al., 2022). *Issatchenkia* spp. had the strongest correlation with pepsin activity (Figure 8) and had the highest abundances among 16S rDNA bacteria and ITS1 fungi. However, pepsin activity was the lowest during the entire conversion of the BSFL gut (Figure 2C).

*Issachenkia* spp. were dominated by *Issatchenkia orientalis* (Figure 3F). *I. orientalis* is also known as *Pichia kudriavzevii* (Kurtzman et al., 2008; Lee et al., 2022). *I. orientalis* is often genetically engineered to produce organic acids (Cao et al., 2020). *I. orientalis* can resist environmental stresses such as high temperature and low pH, and can be used for bioethanol production (Miao et al., 2018; Li et al., 2020). Few studies indicate that *I. orientalis* possesses proteolytic enzyme capacity. We used the KEGG database^6^ to annotate protein data^7^ from the representative genome of *I. orientalis* (Douglass et al., 2018). Annotation results showed that *I. orientalis* lacked proteolytic enzyme proteins such as pepsin (Supplementary Figure S8).

*Issatchenkia orientalis* is unable to complement BSFL with proteolytic enzymes (trypsin, peptidase, pepsin). In our study, bacteria and fungi are mostly negatively correlated, but fungal *Issatchenkia* spp. and bacterial *Lactobacillus* spp. were positively correlated (Figure 4). *Lactobacillus* spp. was highly correlated with all proteolytic enzyme activities (Figure 8). *I. orientalis* can enhance the enzymatic activities (trypsin and peptidase) of *Lactobacillus brevis* and *Lactobacillus plantarum* (Mugula et al., 2003). It is also possible that *I. orientalis* promoted BSFL protein digestion in other, as yet uncharacterized, ways.

Gut microbes play an important role in the nutritional regulation of insects. Gut microbes can influence nutrient sensing signaling pathways in the insect host (Storelli et al., 2011). Some gut microbes can promote amino acid harvest in *Drosophila* (Yamada et al., 2015). In terms of protein metabolism, the degradation of proteins is fundamental. Proteins in insect foods are degraded to polypeptides or amino acids by the action of proteases. To achieve efficient digestion, the proteases secreted by the midgut require a suitable pH. Insect gut pH plays an important role in the digestion of ingested food (Banerjee et al., 2022).

The proteases of insects are mostly trypsin (Sui et al., 2009). Strong trypsin activity has previously been found in the BSFL gut (Kim et al., 2011). Gut microbes may promote BSFL to secrete trypsin, but there is no direct evidence for the role played by gut microbes. The present study provides preliminary evidence that gut microbes elevate trypsin activity in BSFL gut.

Our study showed that gut microbes enhanced the ability of BSFL to degrade proteins. Thus, there is a positive effect on increasing the efficiency with which BSFL converts food protein into insect proteins. BSFL is an edible insect with access to a wide range of foods, and is often used as a protein supplement for animal feed such as fisheries and poultry. We isolated a strain of fungus, *I. orientalis*, from the BSFL gut, which can also be used to treat different foods by BSFL in the future. The more efficient harvest of BSFL insect protein has important implications for alleviating animal feed protein shortage. It is also of great significance for alleviating the human food crisis.

## 5. Conclusion

This study showed that gut microbiota promoted BSFL degradation of casein in artificial diets. *Pseudomonas* spp., *Orbus* spp., and *Campylobacter* spp. were strongly correlated with amino acid metabolism. *Dysgonomonas* spp. had a strong correlation with protein digestion and absorption. *Issatchenkia* spp. was strongly correlated with pepsin activity. *Campylobacter* spp.*, Pediococcus* spp., and *Lactobacillus* spp. were strongly correlated with trypsin activity. *Lactobacillus* spp. and *Bacillu*s spp. were strongly correlated with peptidase activity. BSFL gut microbes such as *Issatchenkia* spp. may promote proteolytic enzyme activity and improve the degradation rate of proteins. BSFL gut microbiota in protein digestion and absorption appear to represent a division of labor. The gut microbiota aid in protein degradation. These results demonstrate that the BSFL gut microbes enhance host protein digestibility.

## Data availability statement

The raw data of the 16S rDNA and the ITS1 amplicon presented in the study are deposited in the NCBI repository, accession number PRJNA877216.

## Author contributions

YY involved in design research and finalized the manuscript, and performed the research. JiaZ and FZ participated part of the experiment and data analysis. MF contributed to aseptic equipment construction. YY, JinZ, and JibZ wrote the paper. MC, LZ, FH, and ZY provided input in experimental design as well as data analysis. All authors contributed to the article and approved the submitted version.

## Funding

This work was supported by the Key Technology R&D Program of Hubei Province, China (2021BBA258), the Major Project of Hubei Hongshan Laboratory (2022hszd013), the Fundamental Research Funds for the Central Universities (2662020SKPY002), and National Key Technology R&D Program of China (2018YFD0500203).

## Conflict of interest

The authors declare that the research was conducted in the absence of any commercial or financial relationships that could be construed as a potential conflict of interest.

## Publisher’s note

All claims expressed in this article are solely those of the authors and do not necessarily represent those of their affiliated organizations, or those of the publisher, the editors and the reviewers. Any product that may be evaluated in this article, or claim that may be made by its manufacturer, is not guaranteed or endorsed by the publisher.

## Footnotes

^1^ http://cloud.allwegene.com/

^2^ http://www.cloudtutu.com/

^4^ https://www.bioinformatics.com.cn

^5^ https://www.genome.jp/kegg/mapper/reconstruct.html

^6^ https://www.kegg.jp/blastkoala/

^7^ https://ftp.ncbi.nlm.nih.gov/genomes/all/GCF/003/054/445/GCF_003054445.1_ASM305444v1/GCF_003054445.1_ASM305444v1_protein.faa.gz
