# Supplementary material for: Enhanced protein degradation by black soldier fly larvae (Hermetia illucens L.) and its gut microbes
Source: Front Microbiol. 2023 Jan 10;13:1095025. doi: 10.3389/fmicb.2022.1095025 (PMC9871565; doi:10.3389/fmicb.2022.1095025)
Supplement: Supplementary file 7 [file Data_Sheet_1.docx]

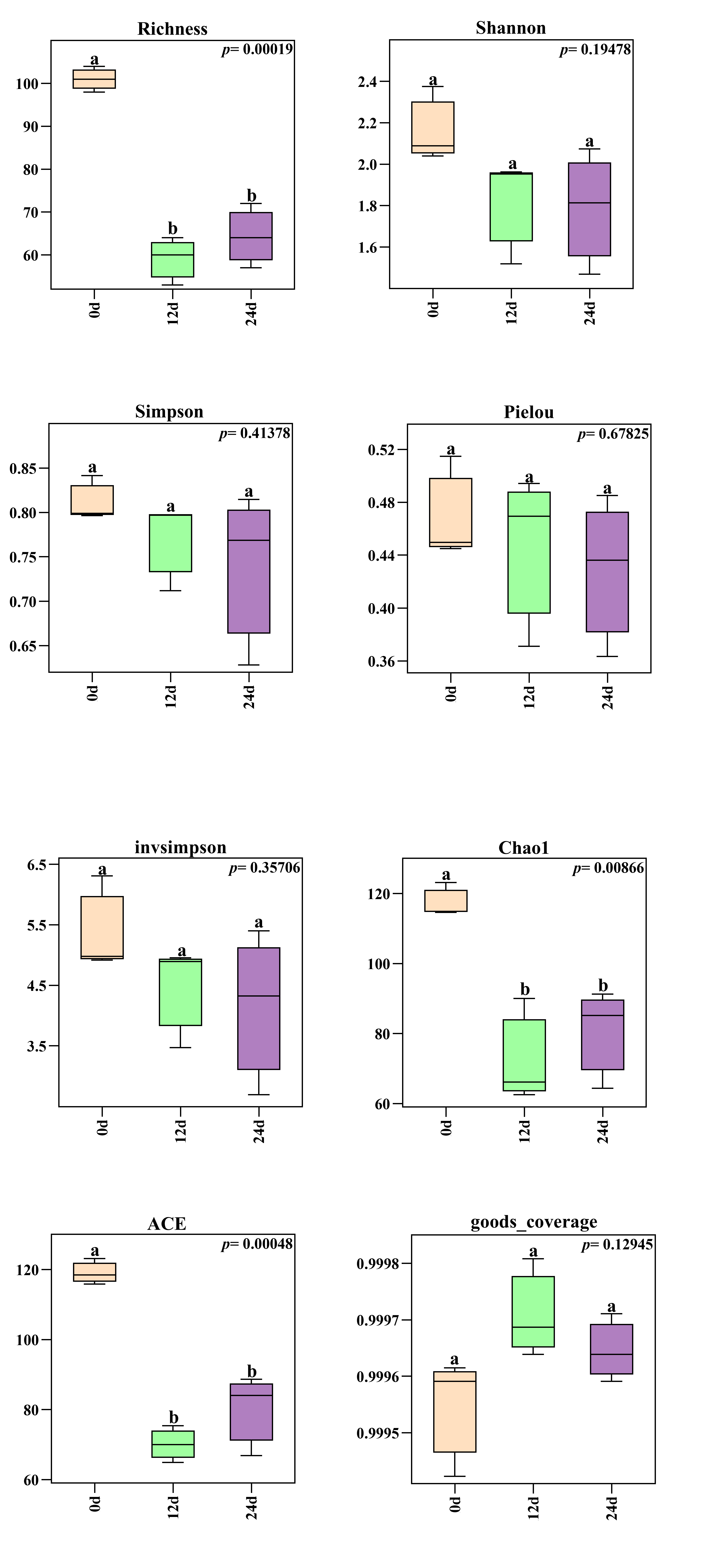


Fig. S1 Gnotobiotic BSFL gut16S-rDNA amplicons α Diversity, data were analyzed by means of one-way ANOVAs and Tukey post hoc comparisons. The overall conversion process *P* value are displayed at the top of each graph. Significant differences (*P* < 0.05) across conversion process are indicated with lowercase letters


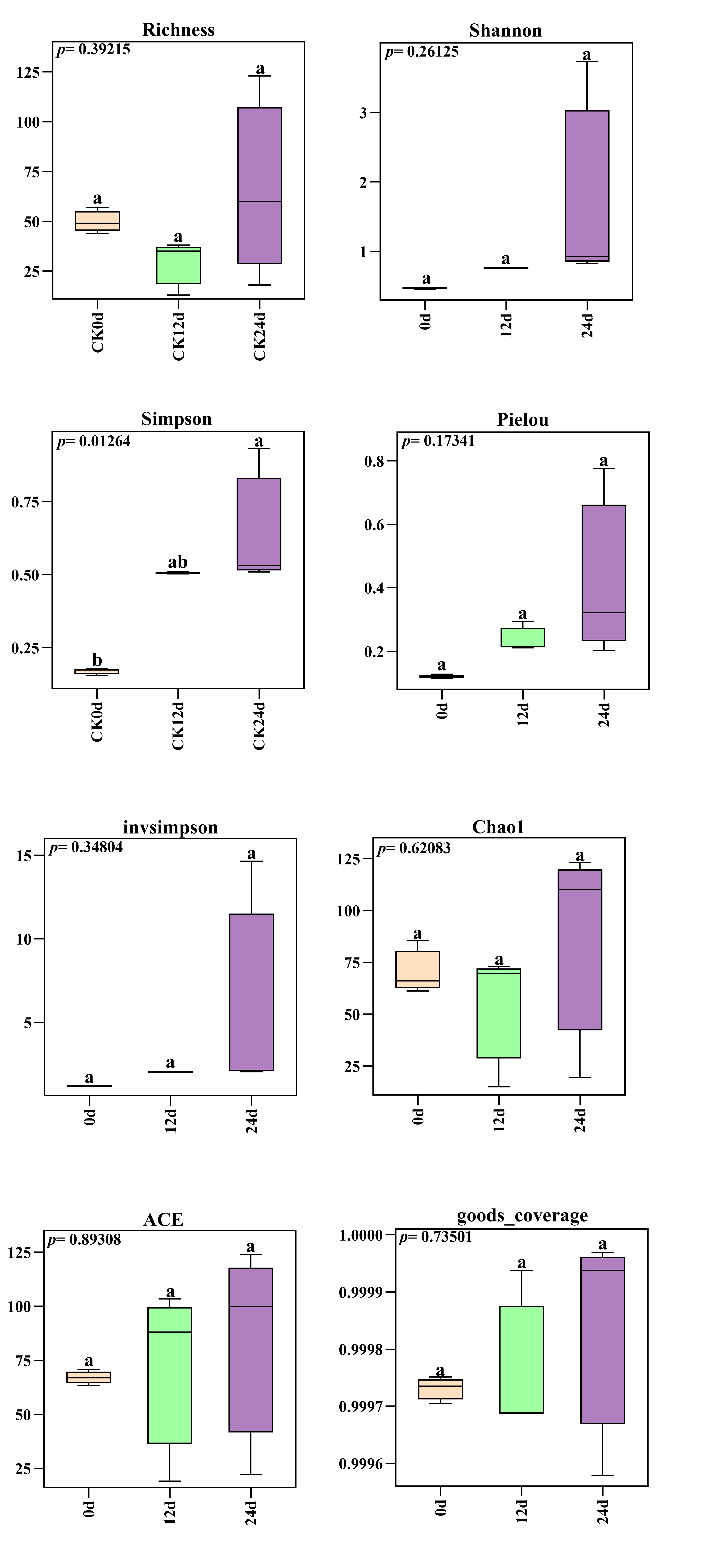


Fig. S2 Gnotobiotic BSFL gut ITS amplicons α Diversity, data were analyzed by means of one-way ANOVAs and Tukey post hoc comparisons. The overall conversion process *P* value are displayed at the top of each graph. Significant differences (*P* < 0.05) across conversion process are indicated with lowercase letters


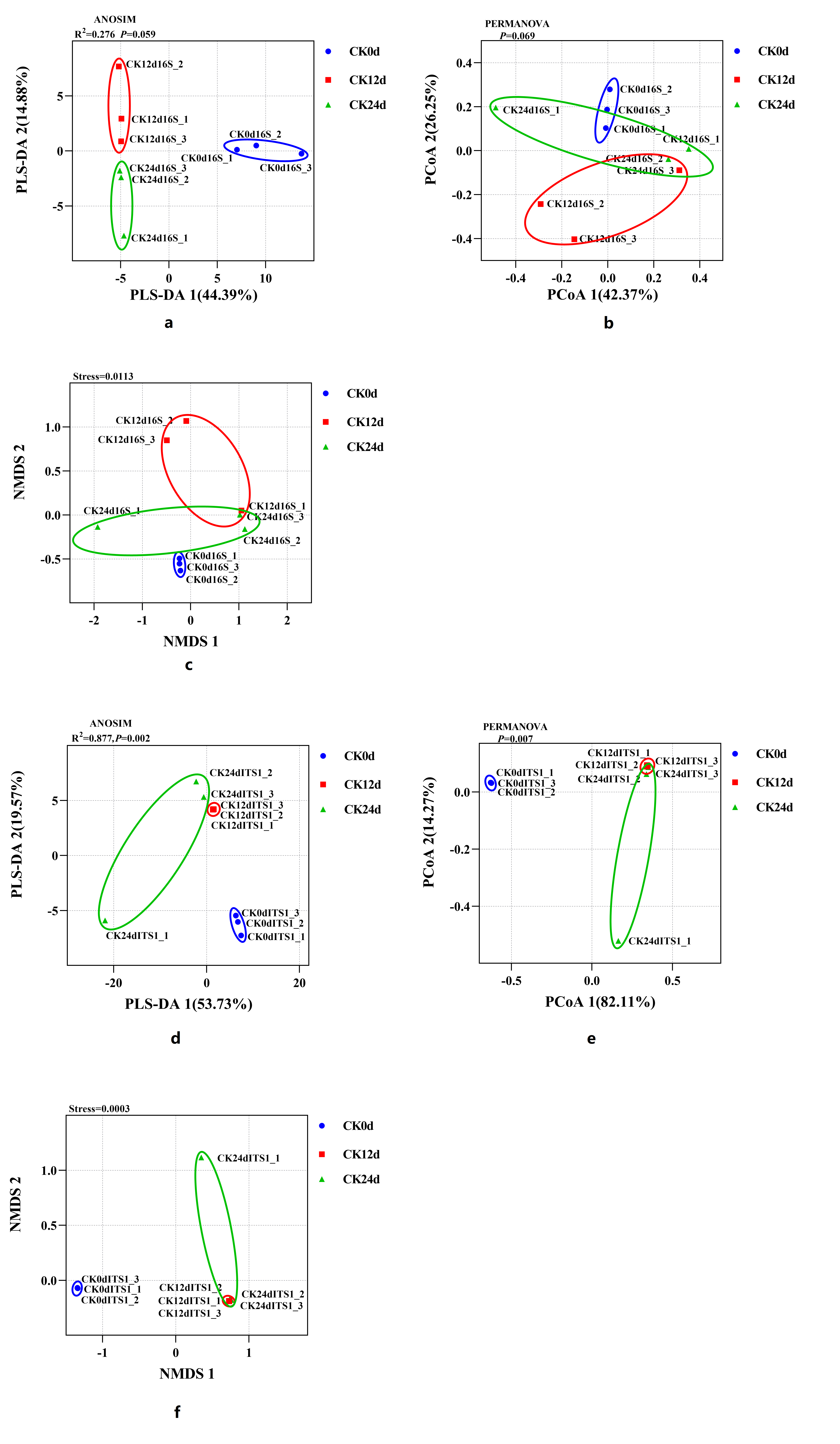


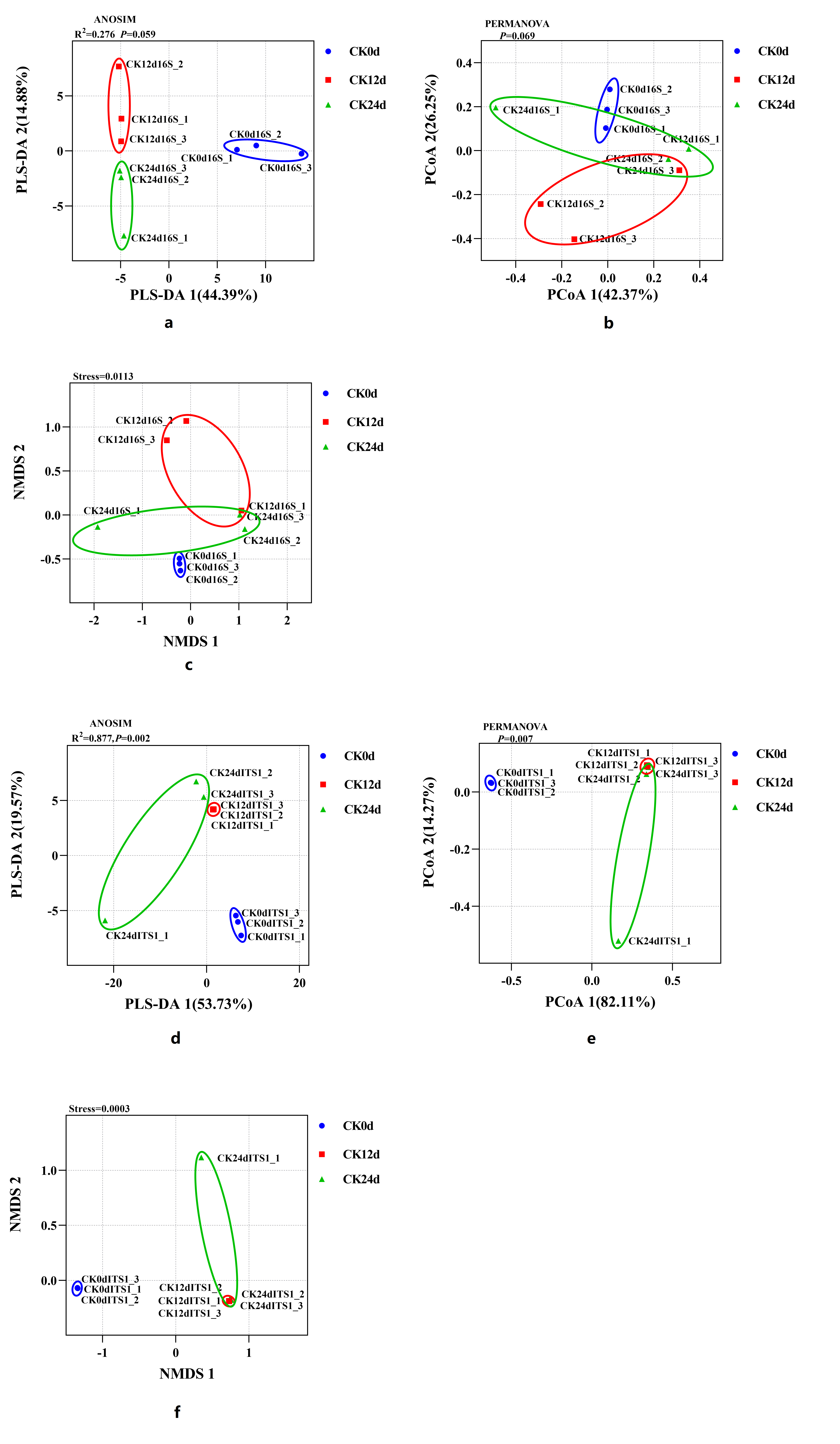


Fig. S3 Gnotobiotic BSFL gut Gut microbiota amplicons β Diversity, 16S-rDNA sequences: a-c; ITS sequences: d-f


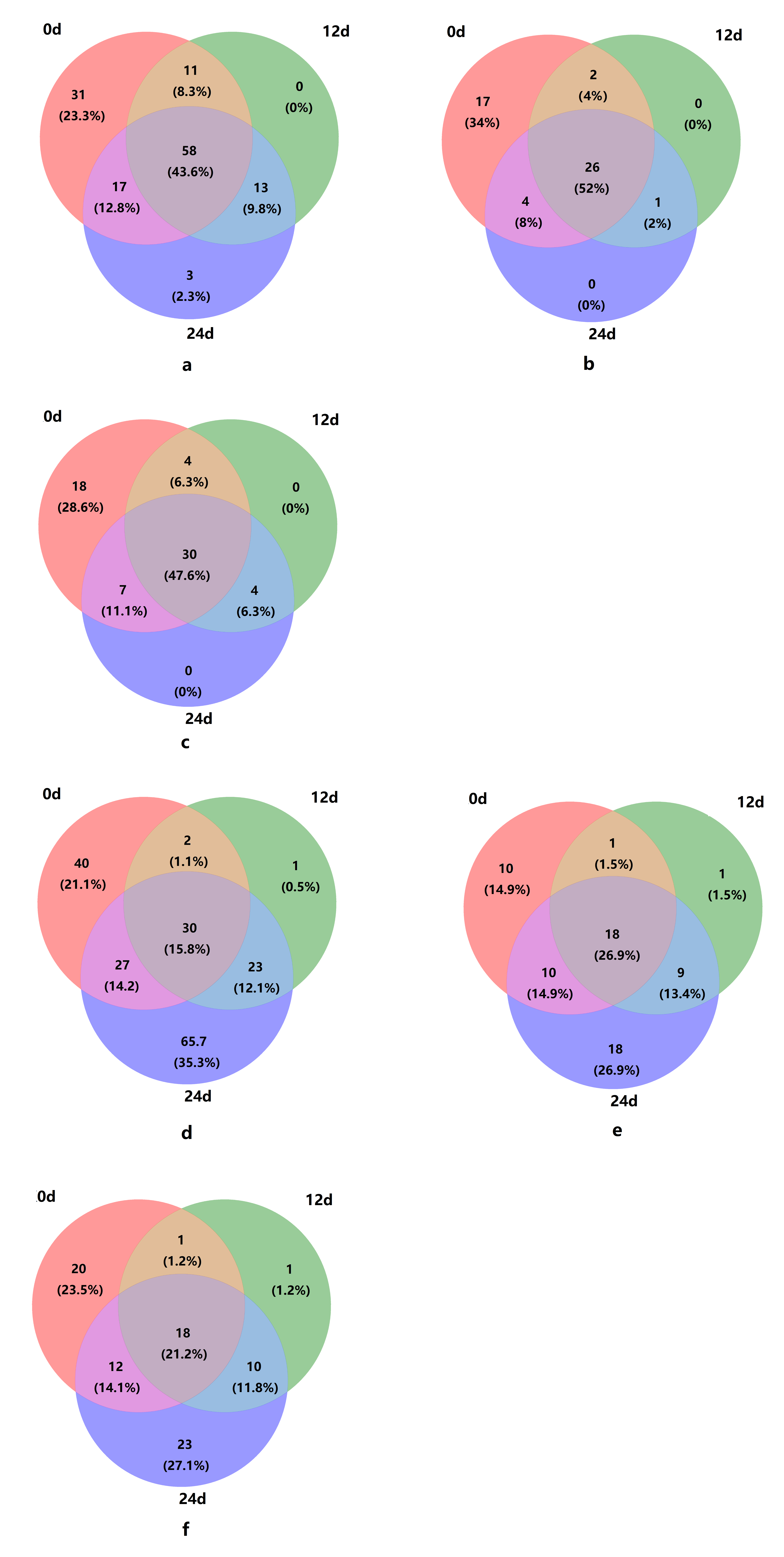


Fig. S4 Venn diagram of Gnotobiotic BSFL gut microbiota amplicons, 16S rDNA sequences: a- OTU level, b- genus level, c- species level; ITS sequences: d- OTU level, e- genus level, f- species level


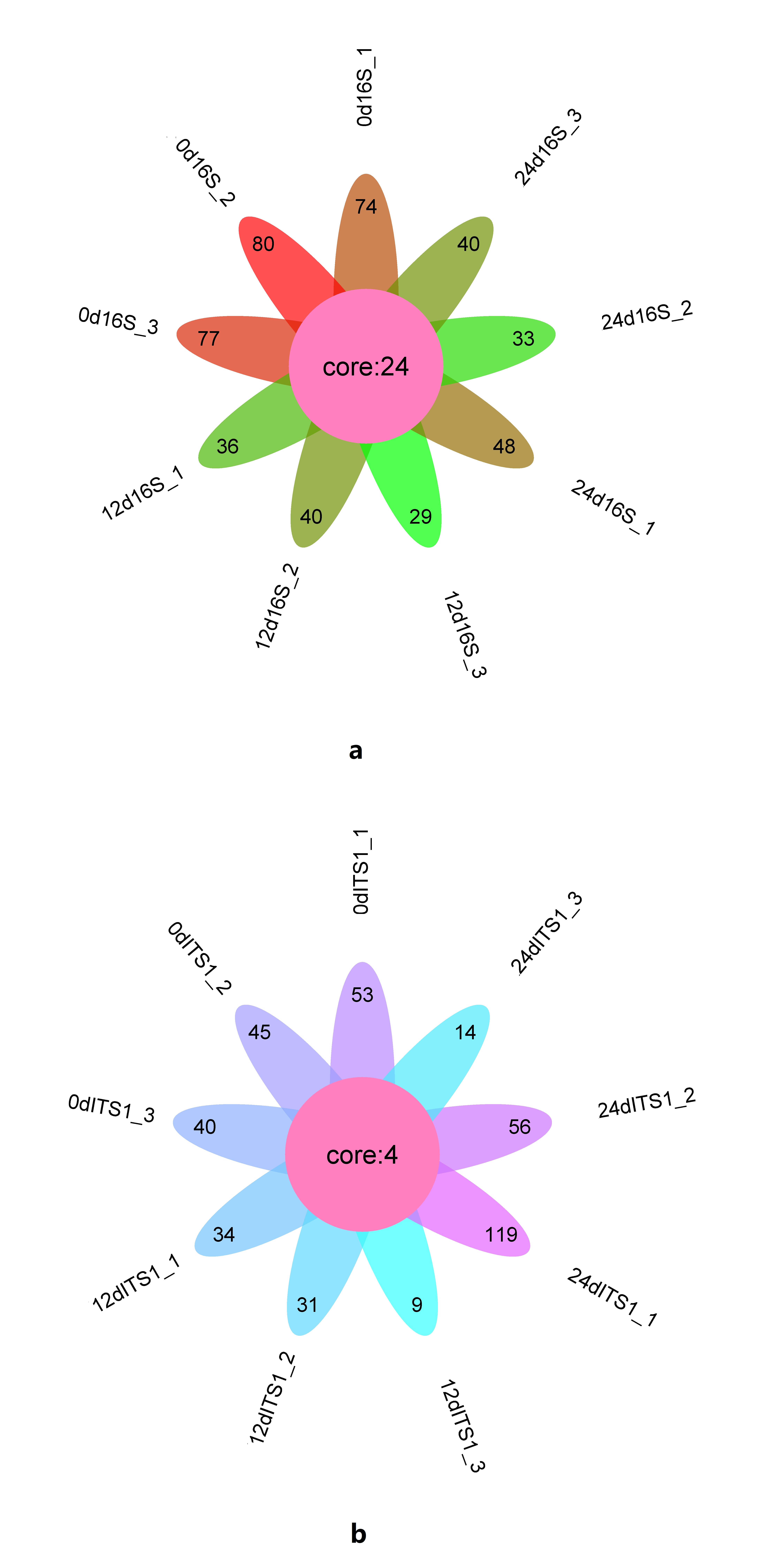


Fig. S5 Flower diagram of gnotobiotic BSFL gut samples at OTU level, a: 16S rDNA sequence; b: ITS1 sequences


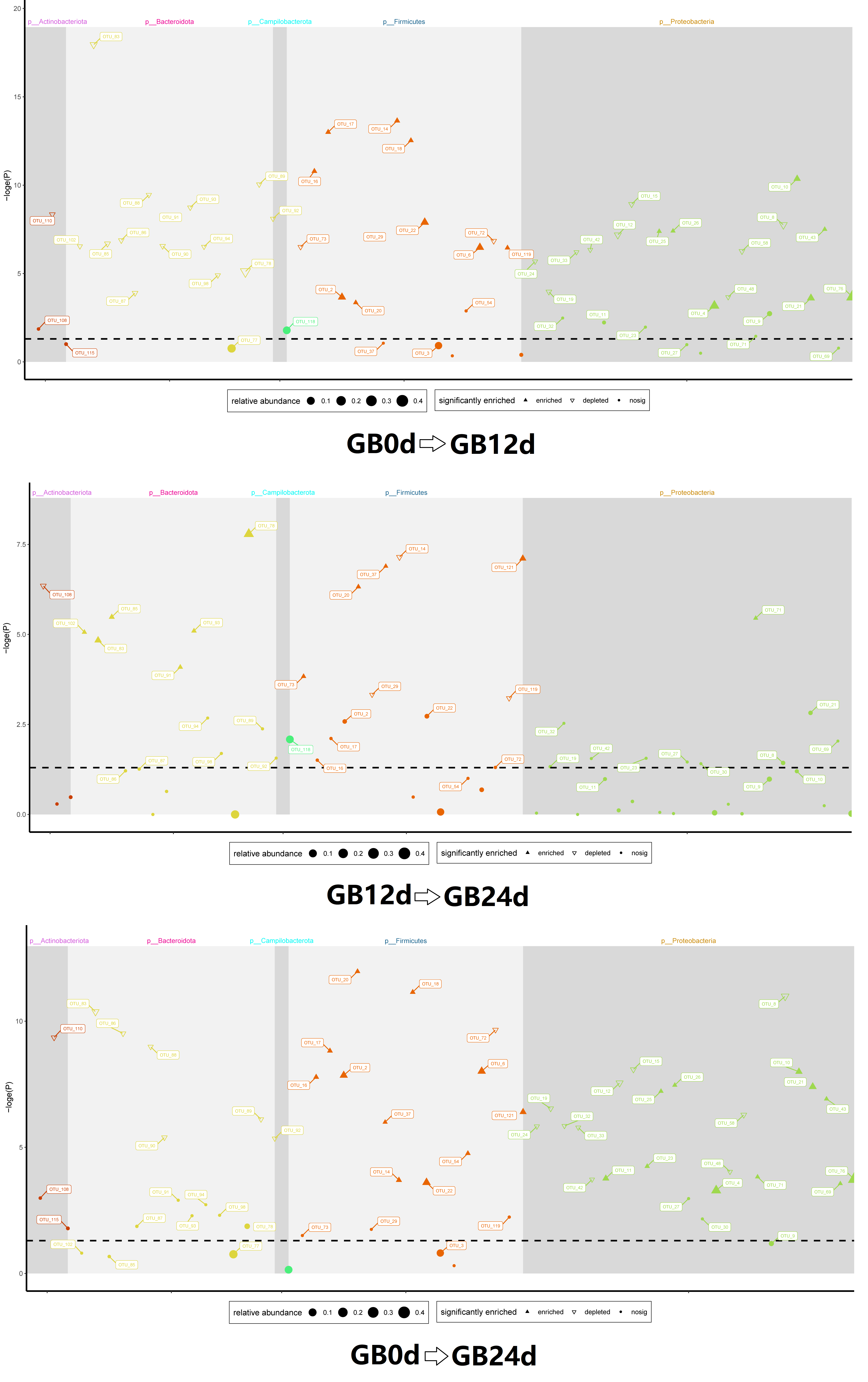
Fig. S6 Manhattan plot of gnotobiotic BSFL gut 16S rDNA amplicons


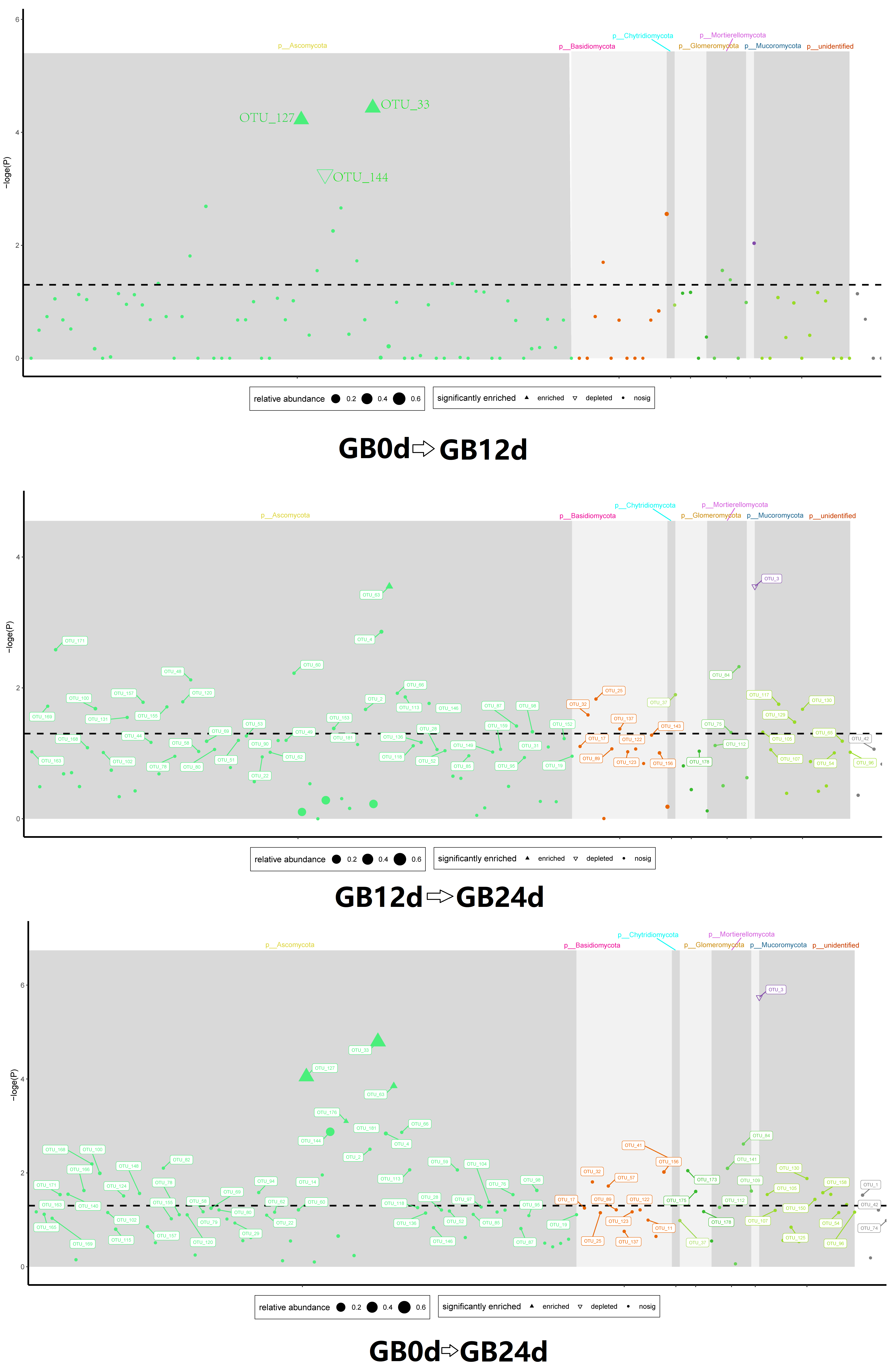


Fig. S7 Manhattan plot of gnotobiotic BSFL gut ITS1 amplicons


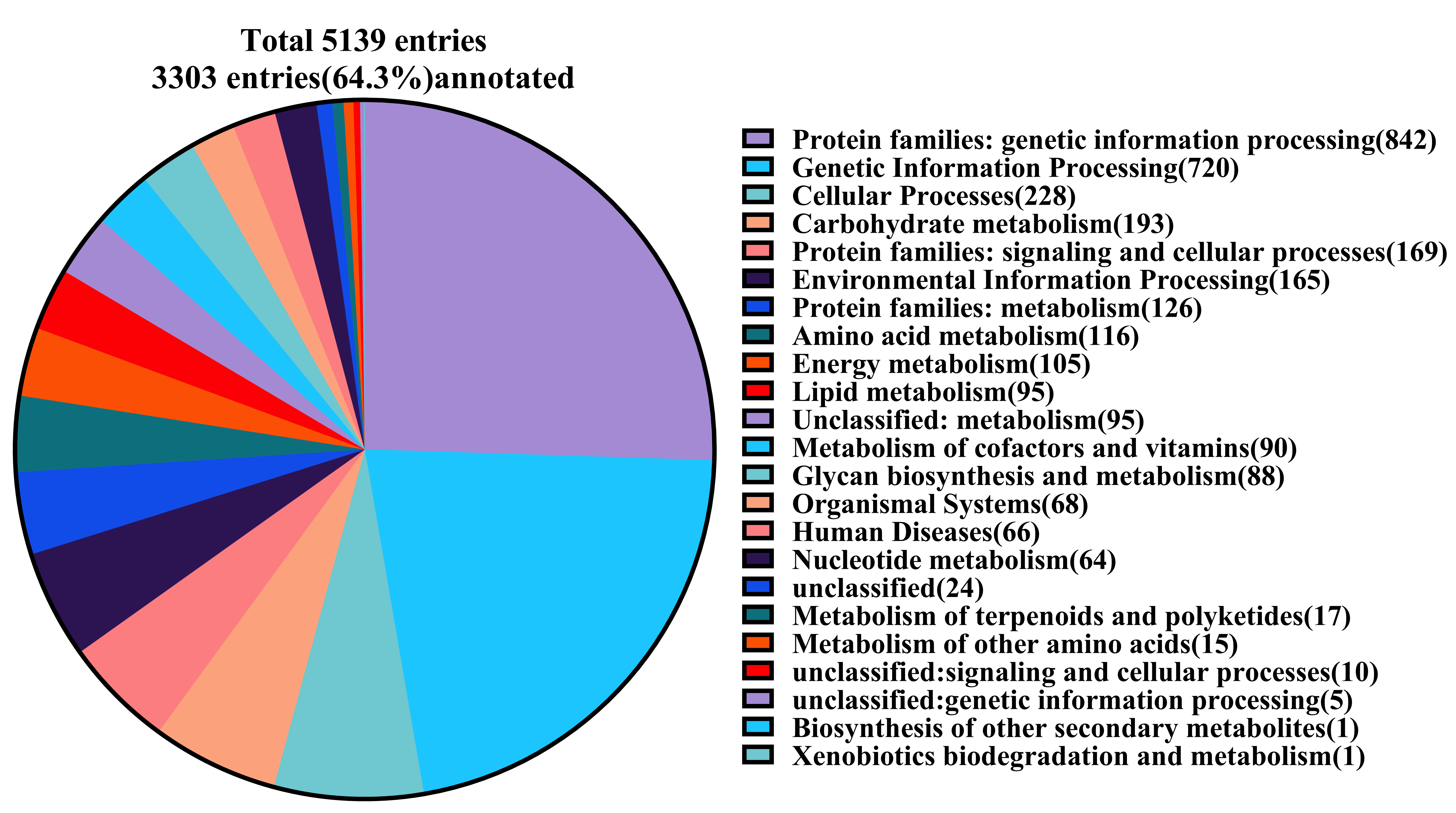


Fig. S8 KEGG annotation of the *Pichia kudriavzevii* CBS573 protein data

| Classification | Component | Content | Dry matter content (%) |
| --- | --- | --- | --- |
| Base composition | Pure water / g | 92.5 | - |
|  | Casein / g | 25 | 47.5 |
|  | Glucose / g | 25 | 47.5 |
|  | Agar/g | 1.5 | 3 |
| Inorganic salt solutions | CaCl_2_(127g/L) /μL | 100 | - |
|  | CuSO_4_**·**5H_2_O(2.5g/L) /μL | 100 | - |
|  | FeSO_4_**·**7H_2_O(25g/L) /μL | 100 | - |
|  | MgSO_4_(250g/L)/ μL | 100 | - |
|  | MnCl_2_**·**4H_2_O(1g/L) /μL | 100 | - |
|  | ZnSO_4_**·**7H_2_O(25g/L)/ μL | 100 | - |
| Other components | Cholesterol (100 mg/mL Chloroform)/ ml | 2.3 | 0.4 |
|  | Vitamin C / g | 0.55 | 1 |
|  | Multivitamins / g | 0.36 | 0.7 |
|  | Solution yeast extract (0.2g/mL)/mL | 10 | 3.8 |

Table S1 Formula of artificial feed

Table S2 Formula of artificial feed Multivitamins solution formula

| Component | Content(mL) |
| --- | --- |
| Vitamin B1(100 mg/mL) | 0.05 |
| Vitamin B2 (0.2 mg/mL) | 25 |
| Vitamin B3 (1.25 mg/mL) | 16.3 |
| Vitamin B5 (50 mg/mL) | 0.21 |
| Vitamin B6 (50 mg/mL) | 0.11 |
| Vitamin B9 (50 mg/mL 1 mol/L NaOH) | 0.11 |
| Vitamin H (80 mg/100mL ethanol) | 0.25 |
| Inositol (50 mg/mL) | 1.1 |
| Para aminobenzoic acid (50 mg/mL ethanol) | 0.11 |
| Choline chloride (1g/mL) | 0.26 |

Table S3 Sterilization method of artificial feed

| Component | Sterilization method |
| --- | --- |
| casein | 115℃，20 minutes |
| Glucose +agar+ water(50g) |  |
| Vitamin B2 |  |
| Vitamin B3 |  |
| Vitamin C |  |
| Solution yeast extract | 121℃，20 minutes |
| Vitamin B1 | 0.22 μm Filtration membrane |
| Vitamin B5 |  |
| Vitamin B6 |  |
| Vitamin B9 |  |
| Vitamin H |  |
| Inositol |  |
| Para aminobenzoic acid |  |
| Choline chloride |  |
| Cholesterol |  |
| Inorganic salt solutions |  |

Table S4 Reagents used for artificial diets

| Reagent name | Cat. No. | Specification | Brand | Storage conditions |
| --- | --- | --- | --- | --- |
| Casein | C8200 | 500g | Solarbio | Room temperature |
| Glucose | 1179GR500 | 500g | Biofroxx |  |
| Agar | 9002-18-0 | 500g | Biofroxx |  |
| YEAST EXTRACT | LP0021 | 500g | OXOID |  |
| cholesterol | BS922 | 25g | biosharp |  |
| Vitamin B1 | V8020 | 10g | Solarbio |  |
| Vitamin B2 | V8060 | 25g |  |  |
| Vitamin B3 | N8060 | 25g |  |  |
| Vitamin B5 | D8170 | 25g |  |  |
| Vitamin B6 | V8030 | 5g |  |  |
| Vitamin B9 | F8090 | 5g |  | 2-8℃ |
| Vitamin H | IB0250 | 10mg |  |  |
| Inositol | I8050 | 100g |  | Room temperature |
| Choline chloride | G8330 | 100g |  |  |
| Para aminobenzoic acid | A9690 | 25g |  |  |
| Vitamin C | 10004016 | 100g | Sinopharm | Room temperature（Keep In Dark Place） |
| CaCl_2_ | 10005861 | 500g |  | Room temperature |
| CuSO_4_·5H_2_O | 10008218 | 500g |  |  |
| FeSO_4_·7H_2_O | 10012118 | 500g |  |  |
| MgSO_4_ | 20025118 | 500g |  |  |
| MnCl_2_·4H_2_O | 20026118 | 500g |  |  |
| ZnSO_4_·7H_2_O | 10024018 | 500g |  |  |

Table S5 Gut proteolytic enzyme activity for individual GB samples

| ID | Trypsin activity（U/mg prot） | Peptidase activity（U/mg prot） | Pepsin activity（U/mg prot） |
| --- | --- | --- | --- |
| GB-0d_1 | 898.1714 | 116.807 | 0.023764 |
| GB-0d_2 | 1047.867 | 109.2042 | 0.115067 |
| GB-0d_3 | 1347.257 | 107.8219 | 0.0863 |
| GB-12d_1 | 3198.625 | 216.1893 | 4.252051 |
| GB-12d_2 | 4051.592 | 203.9699 | 3.910353 |
| GB-12d_3 | 3411.867 | 236.8683 | 3.165939 |
| GB-24d_1 | 589.0509 | 131.8812 | 0.07767 |
| GB-24d_2 | 785.4012 | 86.06985 | 0.091019 |
| GB-24d_3 | 1178.102 | 111.0579 | 0.087379 |
| GF-0d_1 | 181.689 | 84.561 | 0.096 |
| GF-0d_2 | 726.757 | 100.416 | 0.028 |
| GF-0d_3 | 545.068 | 93.809 | 0.063 |
| GF-12d_1 | 1768.672 | 77.390 | 0.126 |
| GF-12d_2 | 2829.875 | 167.922 | 0.118 |
| GF-12d_3 | 2122.406 | 159.161 | 0.110 |
| GF-24d_1 | 655.051 | 51.368 | 0.096 |
| GF-24d_2 | 982.576 | 67.655 | 0.050 |
| GF-24d_3 | 655.051 | 50.115 | 0.057 |

Table S6 Core microbiota of gnotobiotic sample for 16S rDNA amplicons

| Species level | Genus level | OTU Level |
| --- | --- | --- |
| *s__metagenome* | *g__Dysgonomonas* | OTU_10 |
| *s__uncultured_bacterium* | *g__uncultured* | OTU_106 |
| *s__Lactobacillus_plantarum* | *g__Vagococcus* | OTU_108 |
| *s__uncultured_Actinomycetales_bacterium* | *g__Cronobacter* | OTU_11 |
| *s__Morganella_morganii* | *g__Salana* | OTU_110 |
| *s__Enterococcus_moraviensis_ATCC_BAA-383* | *g__Proteus* | OTU_115 |
| *s__Enterococcus_ureilyticus* | *g__Actinomyces* | OTU_118 |
| *s__Cronobacter_dublinensis_subsp._lactaridi_LMG_23825* | *g__Comamonas* | OTU_12 |
| *s__Lactobacillus_rhamnosus_CRL1505* | *g__Providencia* | OTU_120 |
| *s__Heliconius_numata_bicoloratus* | *g__Citrobacter* | OTU_121 |
| *s__Exiguobacterium_mexicanum* | *g__Enterococcus* | OTU_122 |
| *s__Arsenophonus_endosymbiont_str._Hangzhou_of_Nilaparvata_lugens* | *g__Campylobacter* | OTU_14 |
| *s__Comamonas_kerstersii* | *g__Morganella* | OTU_15 |
| *s__Escherichia_coli* | *g__Lactobacillus* | OTU_16 |
| *s__Klebsiella_pneumoniae_BIDMC_21* | *g__Klebsiella* | OTU_17 |
| *s__Proteus_mirabilis_WGLW4* | *g__Dysgonomonas_sp._BGC7* | OTU_19 |
| *s__human_gut_metagenome* | *g__Escherichia-Shigella* | OTU_2 |
| *s__uncultured_organism* | *g__Erysipelothrix* | OTU_20 |
| *s__Dysgonomonas_gadei_ATCC_BAA-286* | *g__unidentified* | OTU_21 |
| *s__Enterococcus_termitis* | *g__Pseudomonas* | OTU_22 |
| *s__bacterium_NLAE-zl-C91* | *g__Orbus* | OTU_24 |
| *s__Lactobacillus_camelliae* | *g__Arsenophonus* | OTU_27 |
| *s__unidentified* | *g__Yersinia* | OTU_3 |
| *s__uncultured_Providencia_sp.* | *g__Gilliamella* | OTU_30 |
| *s__Lactobacillus_brevis* | *g__Cosenzaea* | OTU_35 |
| *s__Heliconius_timareta_timareta* | *g__Bacillus* | OTU_36 |
| *s__Lactobacillus_crustorum_JCM_15951* |  | OTU_4 |
| *s__uncultured_gamma_proteobacterium* |  | OTU_48 |
| *s__Cosenzaea_myxofaciens_ATCC_19692* |  | OTU_54 |
| *s__Morganella_morganii_IS15* |  | OTU_58 |
|  |  | OTU_6 |
|  |  | OTU_63 |
|  |  | OTU_69 |
|  |  | OTU_72 |
|  |  | OTU_73 |
|  |  | OTU_76 |
|  |  | OTU_77 |
|  |  | OTU_78 |
|  |  | OTU_79 |
|  |  | OTU_8 |
|  |  | OTU_80 |
|  |  | OTU_81 |
|  |  | OTU_82 |
|  |  | OTU_83 |
|  |  | OTU_85 |
|  |  | OTU_86 |
|  |  | OTU_87 |
|  |  | OTU_9 |
|  |  | OTU_90 |
|  |  | OTU_98 |
|  |  | OTU_119 |
|  |  | OTU_18 |
|  |  | OTU_23 |
|  |  | OTU_29 |
|  |  | OTU_38 |
|  |  | OTU_45 |
|  |  | OTU_52 |
|  |  | OTU_55 |

Table S7 Core microbiota of gnotobiotic sample for ITS1 amplicons

| Species level | Genus level | OTU Level |
| --- | --- | --- |
| *s__Passalora_arctostaphyli* | *g__Mortierella* | OTU_10 |
| *s__Chaetomium_sp* | *g__Trichosporon* | OTU_100 |
| *s__Trichosporon_asahii* | *g__Candida* | OTU_115 |
| *s__Rhizopus_arrhizus* | *g__Issatchenkia* | OTU_117 |
| *s__Archaeorhizomyces_sp* | *g__Chaetomium* | OTU_125 |
| *s__Issatchenkia_orientalis* | *g__Trichoderma* | OTU_127 |
| *s__Aspergillus_flavus* | *g__Sphaerulina* | OTU_134 |
| *s__Lepraria_granulata* | *g__Lepraria* | OTU_136 |
| *s__Trichoderma_sp* | *g__Saccharomycopsis* | OTU_140 |
| *s__Sphaerulina_sp* | *g__Wickerhamomyces* | OTU_142 |
| *s__Saccharomycopsis_fibuligera* | *g__Cryptococcus* | OTU_144 |
| *s__Candida_parapsilosis* | *g__Archaeorhizomyces* | OTU_150 |
| *s__Mortierella_elongata* | *g__unidentified* | OTU_153 |
| *s__Wickerhamomyces_anomalus* | *g__Pyrenochaeta* | OTU_156 |
| *s__unidentified* | *g__Russula* | OTU_165 |
| *s__Candida_tropicalis* | *g__Rhizopus* | OTU_176 |
| *s__Pyrenochaeta_unguis-hominis* | *g__Passalora* | OTU_18 |
| *s__Cryptococcus_sp* | *g__Aspergillus* | OTU_180 |
|  |  | OTU_181 |
|  |  | OTU_2 |
|  |  | OTU_3 |
|  |  | OTU_33 |
|  |  | OTU_35 |
|  |  | OTU_38 |
|  |  | OTU_4 |
|  |  | OTU_41 |
|  |  | OTU_63 |
|  |  | OTU_67 |
|  |  | OTU_69 |
|  |  | OTU_98 |

Table S8 OTU abundances Top 20 of all amplicon (16S rDNA and ITS1)

| ID | phylum | abundance |
| --- | --- | --- |
| *g__Archaeorhizomyces* | *p__Ascomycota* | 2108 |
| *g__Candida* | *p__Ascomycota* | 2203 |
| *g__Aspergillus* | *p__Ascomycota* | 2260 |
| *g__Lactobacillus* | *p__Firmicutes* | 2412 |
| *g__Proteus* | *p__Proteobacteria* | 3001 |
| *g__Actinomyces* | *p__Actinobacteriota* | 3502 |
| *g__Klebsiella* | *p__Proteobacteria* | 3631 |
| *g__Orbus* | *p__Proteobacteria* | 4011 |
| *g__Trichosporon* | *p__Basidiomycota* | 7438 |
| *g__Wickerhamomyces* | *p__Ascomycota* | 8529 |
| *g__Vagococcus* | *p__Firmicutes* | 8765 |
| *g__Providencia* | *p__Proteobacteria* | 14320 |
| *g__uncultured* | *p__Proteobacteria* | 16264 |
| *g__Yersinia* | *p__Proteobacteria* | 18303 |
| *g__Pseudomonas* | *p__Proteobacteria* | 39199 |
| *g__Campylobacter* | *p__Campilobacterota* | 69705 |
| *g__Enterococcus* | *p__Firmicutes* | 74196 |
| *g__Dysgonomonas* | *p__Bacteroidota* | 112188 |
| *g__unidentified* | *p__Ascomycota* | 200680 |
| *g__Issatchenkia* | *p__Ascomycota* | 334125 |

Table S9 All amplicon (16S rDNA and ITS1) abundances TOP20 co-network P value and R value

| ID | ID | P | R |
| --- | --- | --- | --- |
| *g__Archaeorhizomyces* | *g__Dysgonomonas* | 0.044707 | 0.678064 |
| *g__Candida* | *g__Aspergillus* | 0.016088 | 0.76602 |
| *g__Candida* | *g__Lactobacillus* | 9.02E-05 | -0.94958 |
| *g__Candida* | *g__Klebsiella* | 0.023655 | 0.736408 |
| *g__Candida* | *g__Orbus* | 0.001426 | -0.88704 |
| *g__Candida* | *g__Trichosporon* | 0.013882 | 0.776378 |
| *g__Candida* | *g__unidentified* | 0.028819 | 0.719672 |
| *g__Candida* | *g__Issatchenkia* | 0.005795 | -0.82846 |
| *g__Aspergillus* | *g__Lactobacillus* | 0.020202 | -0.749 |
| *g__Aspergillus* | *g__Trichosporon* | 0.009092 | 0.803448 |
| *g__Aspergillus* | *g__uncultured* | 0.025884 | 0.728918 |
| *g__Aspergillus* | *g__Campylobacter* | 0.031425 | -0.71197 |
| *g__Aspergillus* | *g__Issatchenkia* | 0.021026 | -0.74587 |
| *g__Lactobacillus* | *g__Klebsiella* | 0.019142 | -0.75314 |
| *g__Lactobacillus* | *g__Orbus* | 0.017117 | 0.761513 |
| *g__Lactobacillus* | *g__Trichosporon* | 0.013882 | -0.77638 |
| *g__Lactobacillus* | *g__Yersinia* | 0.010445 | 0.794986 |
| *g__Lactobacillus* | *g__Issatchenkia* | 0.011909 | 0.786618 |
| *g__Proteus* | *g__Providencia* | 0.002806 | -0.86193 |
| *g__Actinomyces* | *g__Orbus* | 0.03577 | -0.7 |
| *g__Actinomyces* | *g__Trichosporon* | 0.018354 | 0.756329 |
| *g__Actinomyces* | *g__Wickerhamomyces* | 0.047275 | -0.67229 |
| *g__Actinomyces* | *g__unidentified* | 0.002495 | 0.616667 |
| *g__Klebsiella* | *g__Yersinia* | 0.024554 | -0.73333 |
| *g__Orbus* | *g__unidentified* | 0.015944 | -0.76667 |
| *g__Orbus* | *g__Issatchenkia* | 0.005266 | 0.833333 |
| *g__Trichosporon* | *g__uncultured* | 0.022768 | 0.739522 |
| *g__Trichosporon* | *g__unidentified* | 0.007424 | 0.815155 |
| *g__Trichosporon* | *g__Issatchenkia* | 0.018354 | -0.75633 |
| *g__uncultured* | *g__Enterococcus* | 0.002495 | 0.866667 |
| *g__uncultured* | *g__unidentified* | 0.002495 | 0.866667 |
| *g__Enterococcus* | *g__unidentified* | 0.003705 | 0.85 |
| *g__unidentified* | *g__Issatchenkia* | 0.049867 | -0.66667 |

Table S10 The proteinogenic amino acid metabolism and metabolism of non-protein amino acids associated with BSFL gut microbiota

| KEGG Metabolism | KEGG Metabolic classification |
| --- | --- |
| Alanine, aspartate and glutamate metabolism | Proteinogenic amino acid metabolism |
| Glycine, serine and threonine metabolism |  |
| Valine, leucine and isoleucine degradation |  |
| Valine, leucine and isoleucine biosynthesis |  |
| Lysine biosynthesis |  |
| Lysine degradation |  |
| Arginine and proline metabolism |  |
| Histidine metabolism |  |
| Tyrosine metabolism |  |
| Phenylalanine metabolism |  |
| Tryptophan metabolism |  |
| beta-Alanine metabolism | Metabolism of non-protein amino acids |
| Taurine and hypotaurine metabolism |  |
| Phosphonate and phosphinate metabolism |  |
| Cyanoamino acid metabolism |  |
| D-Glutamine and D-glutamate metabolism |  |
| D-Arginine and D-ornithine metabolism |  |
| D-Alanine metabolism |  |
| Glutathione metabolism |  |

Table S11 16S rDNA amplicon abundances and protein metabolism KEGG enrichment abundance co-network R value and P value

| Target | Source | P | R |
| --- | --- | --- | --- |
| Cyanoamino.acid.metabolism | *g__Campylobacter* | 0.003166 | 0.856875 |
| Protein.digestion.and.absorption | *g__Campylobacter* | 0.038173 | 0.693782 |
| Cyanoamino.acid.metabolism | *g__Dysgonomonas* | 0.005003 | 0.835872 |
| Protein.digestion.and.absorption | *g__Dysgonomonas* | 0.010156 | 0.796725 |
| Alanine..aspartate.and.glutamate.metabolism | *g__Dysgonomonas* | 0.043434 | 0.681005 |
| Cyanoamino.acid.metabolism | *g__Dysgonomonas* | 0.002959 | 0.859727 |
| Protein.digestion.and.absorption | *g__Dysgonomonas* | 0.022467 | 0.740593 |
| Alanine..aspartate.and.glutamate.metabolism | *g__Dysgonomonas* | 0.019942 | 0.75 |
| Taurine.and.hypotaurine.metabolism | *g__Dysgonomonas* | 0.024554 | 0.75 |
| Cyanoamino.acid.metabolism | *g__Dysgonomonas* | 0.006573 | 0.821822 |
| Protein.digestion.and.absorption | *g__Dysgonomonas* | 0.009628 | 0.8 |
| Cyanoamino.acid.metabolism | *g__Pseudomonas* | 0.040589 | 0.687782 |
| Protein.digestion.and.absorption | *g__Pseudomonas* | 0.047184 | 0.672493 |
| Cyanoamino.acid.metabolism | *g__Dysgonomonas* | 0.011512 | 0.788818 |
| Protein.digestion.and.absorption | *g__Dysgonomonas* | 0.022768 | 0.739522 |
| Protein.digestion.and.absorption | *g__uncultured1* | 0.049554 | 0.667336 |
| Lysine.degradation | *g__Orbus* | 0.032793 | 0.708088 |
| Tyrosine.metabolism | *g__Orbus* | 0.012654 | 0.782624 |
| Tryptophan.metabolism | *g__Orbus* | 0.032793 | 0.708088 |
| Taurine.and.hypotaurine.metabolism | *g__Orbus* | 0.047945 | 0.67082 |
| Glycine..serine.and.threonine.metabolism | *g__Pseudomonas* | 0.001305 | 0.889946 |
| Valine..leucine.and.isoleucine.degradation | *g__Pseudomonas* | 0.006108 | 0.825723 |
| Lysine.degradation | *g__Pseudomonas* | 0.001305 | 0.889946 |
| Arginine.and.proline.metabolism | *g__Pseudomonas* | 0.001305 | 0.889946 |
| Histidine.metabolism | *g__Pseudomonas* | 0.00724 | 0.816548 |
| Tyrosine.metabolism | *g__Pseudomonas* | 0.001305 | 0.889946 |
| Phenylalanine.metabolism | *g__Pseudomonas* | 0.001305 | 0.889946 |
| Tryptophan.metabolism | *g__Pseudomonas* | 0.001305 | 0.889946 |
| beta.Alanine.metabolism | *g__Pseudomonas* | 0.001305 | 0.889946 |
| Glycine..serine.and.threonine.metabolism | *g__Pseudomonas* | 0.003076 | 0.858099 |
| Valine..leucine.and.isoleucine.degradation | *g__Pseudomonas* | 0.009111 | 0.803326 |
| Lysine.degradation | *g__Pseudomonas* | 0.003076 | 0.858099 |
| Arginine.and.proline.metabolism | *g__Pseudomonas* | 0.003076 | 0.858099 |
| Histidine.metabolism | *g__Pseudomonas* | 0.003076 | 0.858099 |
| Tyrosine.metabolism | *g__Pseudomonas* | 0.003076 | 0.858099 |
| Phenylalanine.metabolism | *g__Pseudomonas* | 0.003076 | 0.858099 |
| Tryptophan.metabolism | *g__Pseudomonas* | 0.003076 | 0.858099 |
| beta.Alanine.metabolism | *g__Pseudomonas* | 0.003076 | 0.858099 |
| Glycine..serine.and.threonine.metabolism | *g__Orbus* | 0.012732 | 0.782217 |
| Valine..leucine.and.isoleucine.degradation | *g__Orbus* | 0.012732 | 0.782217 |
| Lysine.degradation | *g__Orbus* | 0.012732 | 0.782217 |
| Arginine.and.proline.metabolism | *g__Orbus* | 0.012732 | 0.782217 |
| Histidine.metabolism | *g__Orbus* | 0.004442 | 0.841625 |
| Tyrosine.metabolism | *g__Orbus* | 0.012732 | 0.782217 |
| Phenylalanine.metabolism | *g__Orbus* | 0.012732 | 0.782217 |
| Tryptophan.metabolism | *g__Orbus* | 0.012732 | 0.782217 |
| beta.Alanine.metabolism | *g__Orbus* | 0.012732 | 0.782217 |
| Phosphonate.and.phosphinate.metabolism | *g__Orbus* | 0.016908 | 0.762414 |
| Glycine..serine.and.threonine.metabolism | *g__Pseudomonas* | 0.015944 | 0.766667 |
| Valine..leucine.and.isoleucine.degradation | *g__Pseudomonas* | 0.000236 | 0.933333 |
| Lysine.degradation | *g__Pseudomonas* | 8.76E-05 | 0.95 |
| Arginine.and.proline.metabolism | *g__Pseudomonas* | 0 | 1 |
| Histidine.metabolism | *g__Pseudomonas* | 0.042442 | 0.683333 |
| Tyrosine.metabolism | *g__Pseudomonas* | 0.007225 | 0.816667 |
| Phenylalanine.metabolism | *g__Pseudomonas* | 1.94E-06 | 0.983333 |
| Tryptophan.metabolism | *g__Pseudomonas* | 0.000507 | 0.916667 |
| beta.Alanine.metabolism | *g__Pseudomonas* | 1.94E-06 | 0.983333 |
| Phosphonate.and.phosphinate.metabolism | *g__Pseudomonas* | 0.019942 | 0.75 |
| Lysine.degradation | *g__Orbus* | 0.048229 | 0.670199 |
| Tyrosine.metabolism | *g__Orbus* | 0.012517 | 0.783349 |
| Tryptophan.metabolism | *g__Orbus* | 0.048229 | 0.670199 |
| Taurine.and.hypotaurine.metabolism | *g__Orbus* | 0.001394 | 0.887796 |
| Glutathione.metabolism | *g__Orbus* | 0.033905 | 0.705015 |
| Tyrosine.metabolism | *g__Morganella* | 0.016106 | 0.765942 |
| Taurine.and.hypotaurine.metabolism | *g__Morganella* | 0.000393 | 0.922612 |
| Glutathione.metabolism | *g__Morganella* | 0.027924 | 0.722422 |
| Lysine.degradation | *g__Orbus* | 0.042442 | 0.683333 |
| Tyrosine.metabolism | *g__Orbus* | 0.007225 | 0.816667 |
| Tryptophan.metabolism | *g__Orbus* | 0.029818 | 0.716667 |
| Taurine.and.hypotaurine.metabolism | *g__Orbus* | 0.003705 | 0.85 |
| Glutathione.metabolism | *g__Orbus* | 0.049867 | 0.666667 |
| Tyrosine.metabolism | *g__Providencia* | 0.029711 | 0.716985 |
| Taurine.and.hypotaurine.metabolism | *g__Providencia* | 0.000924 | 0.900603 |
| Glutathione.metabolism | *g__Providencia* | 0.03938 | 0.690754 |
| D.Glutamine.and.D.glutamate.metabolism | *g__uncultured2* | 0.049554 | 0.667336 |
| D.Arginine.and.D.ornithine.metabolism | *g__Pediococcus* | 0.03596 | 0.699497 |
| Valine..leucine.and.isoleucine.biosynthesis | *g__Campylobacter* | 0.049867 | 0.666667 |
| D.Arginine.and.D.ornithine.metabolism | *g__Campylobacter* | 1.94E-06 | 0.983333 |
| D.Alanine.metabolism | *g__Campylobacter* | 0.03577 | 0.7 |
| Valine..leucine.and.isoleucine.biosynthesis | *g__Actinomyces* | 0.002495 | 0.866667 |
| Lysine.biosynthesis | *g__Actinomyces* | 0.005266 | 0.833333 |
| D.Arginine.and.D.ornithine.metabolism | *g__Actinomyces* | 0.001591 | 0.883333 |
| D.Alanine.metabolism | *g__Actinomyces* | 0.000507 | 0.916667 |

Table S12 16S rDNA, ITS1 amplicon abundances and protein digestion enzyme activity abundance co-network R value and P value

| Target | Source | P | R |
| --- | --- | --- | --- |
| Trypsin.activity | *g__Campylobacter* | 0.019942 | 0.75 |
| Trypsin.activity | *g__Lactobacillus* | 0.005555 | 0.830628 |
| Trypsin.activity | *g__Pediococcus* | 0.005553 | 0.830653 |
| Trypsin.activity | *g__Issatchenkia* | 0.03082 | 0.713718 |
| Peptidase.activity | *g__Lactobacillus* | 0.01524 | 0.769881 |
| Peptidase.activity | *g__Lactobacillus* | 0.004802 | 0.837882 |
| Peptidase.activity | *g__Campylobacter* | 0.049867 | 0.666667 |
| Peptidase.activity | *g__Bacillus* | 0.010577 | 0.794198 |
| Pepsin.activity | *g__Lactobacillus* | 0.02783 | 0.722715 |
| Pepsin.activity | *g__Lactobacillus* | 0.029311 | 0.718185 |
| Pepsin.activity | *g__Orbus* | 0.029818 | 0.716667 |
| Pepsin.activity | *g__Lactobacillus* | 0.010156 | 0.796725 |
| Pepsin.activity | *g__Morganella* | 0.032498 | 0.708918 |
| Pepsin.activity | *g__unidentified* | 0.024554 | 0.733333 |
| Pepsin.activity | *g__Issatchenkia* | 0.015944 | 0.766667 |
| Pepsin.activity | *g__Issatchenkia* | 0.005034 | 0.835573 |
| Pepsin.activity | *g__Issatchenkia* | 0.016233 | 0.765375 |
